# High-resolution analysis of the human T-cell receptor repertoire

**DOI:** 10.1038/ncomms9081

**Published:** 2015-09-01

**Authors:** Eliana Ruggiero, Jan P. Nicolay, Raffaele Fronza, Anne Arens, Anna Paruzynski, Ali Nowrouzi, Gökçe Ürenden, Christina Lulay, Sven Schneider, Sergij Goerdt, Hanno Glimm, Peter H. Krammer, Manfred Schmidt, Christof von Kalle

**Affiliations:** 1 Department of Translational Oncology, National Center for Tumor Diseases and German Cancer Research Center, Im Neuenheimer Feld 280, 69120 Heidelberg, Germany; 2 Division of Immunogenetics, German Cancer Research Center, Im Neuenheimer Feld 280, 69120 Heidelberg, Germany; 3 Department of Dermatology, Venereology and Allergology, University Medical Center, Theodor-Kutzer-Ufer 1-3, 68167 Mannheim, Germany; 4 Institute for Clinical Chemistry, University Medical Center, Ruprecht-Karls-University of Heidelberg, Theodor-Kutzer-Ufer 1-3, 68167 Mannheim, Germany

## Abstract

Unbiased dissection of T-cell receptor (TCR) repertoire diversity at the nucleotide level could provide important insights into human immunity. Here we show that TCR ligation-anchored-magnetically captured PCR (TCR-LA-MC PCR) identifies TCR α- and β-chain diversity without sequence-associated or quantitative restrictions in healthy and diseased conditions. TCR-LA-MC PCR identifies convergent recombination events, classifies different stages of cutaneous T-cell lymphoma *in vivo* and demonstrates TCR reactivation after *in vitro* cytomegalovirus stimulation. TCR-LA-MC PCR allows ultra-deep data access to both physiological TCR diversity and mechanisms influencing clonality in all clinical settings with restricted or distorted TCR repertoires.

## Abstract (web summary)

Immune system diversity is generated by V(D)J recombination, leading to clonal T-cell lineages. Here the authors investigate the events leading to T-cell diversity through the use of a modified PCR technique combined with deep sequencing.

## Graphical abstract

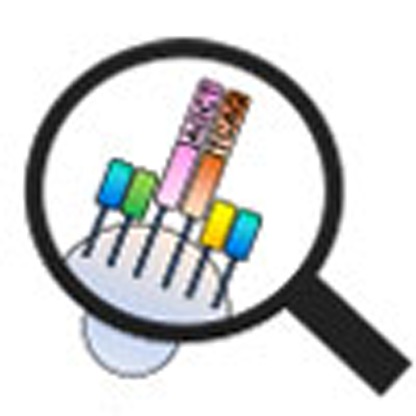

The naive TCR repertoire is assembled in the thymus and formed by somatic rearrangements of non-contiguous genes belonging to the variable (*V*), diversity (*D*; only for the β- and δ-chain) and joining (*J*) families (combinatorial diversity). These are adjacent to the constant (*C*) gene in the heterodimeric α–β or γ–δ TCR. Further random insertion and deletion of nucleotides at the rearrangement positions create junctional diversity of the highly variable complementarity-determining region 3 (CDR3 region). The CDR3 region is unique for every T-cell clone and encodes the receptor portion that makes the majority of TCR contacts with antigenic peptides bound by the major histocompatibility complex. The frequency of a specific CDR3 sequence indicates the abundance of its T-cell clone. Comprehensive and unbiased TCR deep sequencing could provide a representative and quantitative repertoire analysis of specific cellular immunity. Outside of rapid amplification of cDNA ends (RACE) PCR^1^,^2^,^3^,^4^,^5^, current DNA-based TCR-sequencing technologies require extensive primer multiplexing^6^,^7^,^8^,^9^ and are almost exclusively focused on the β-chain repertoire. To obtain the highest possible resolution at high specificity, we have made use of previously established PCR-based methods: ligation-anchored (LA) PCR^10^,^11^ and non-restrictive linear amplification-mediated PCR^12^,^13^,^14^, and developed TCR-LA-MC PCR as a new sequencing technology for the immunogenetic characterization of normal and reduced clonality in the α- and β-chain TCR repertoire. Here we show the identification of the events leading to the generation of the TCR diversity in healthy donors (HDs), discrimination of different stages of cutaneous T-cell lymphoma *in vivo* and monitoring of the reactivation of specific TCR clones after *in vitro* stimulation with peptide pools of two immunodominant cytomegalovirus (CMV) antigens, providing high-resolution characterization of TCR clonality repertoires.

## Results

### T-cell receptor direct sequencing

**Figure 1:**
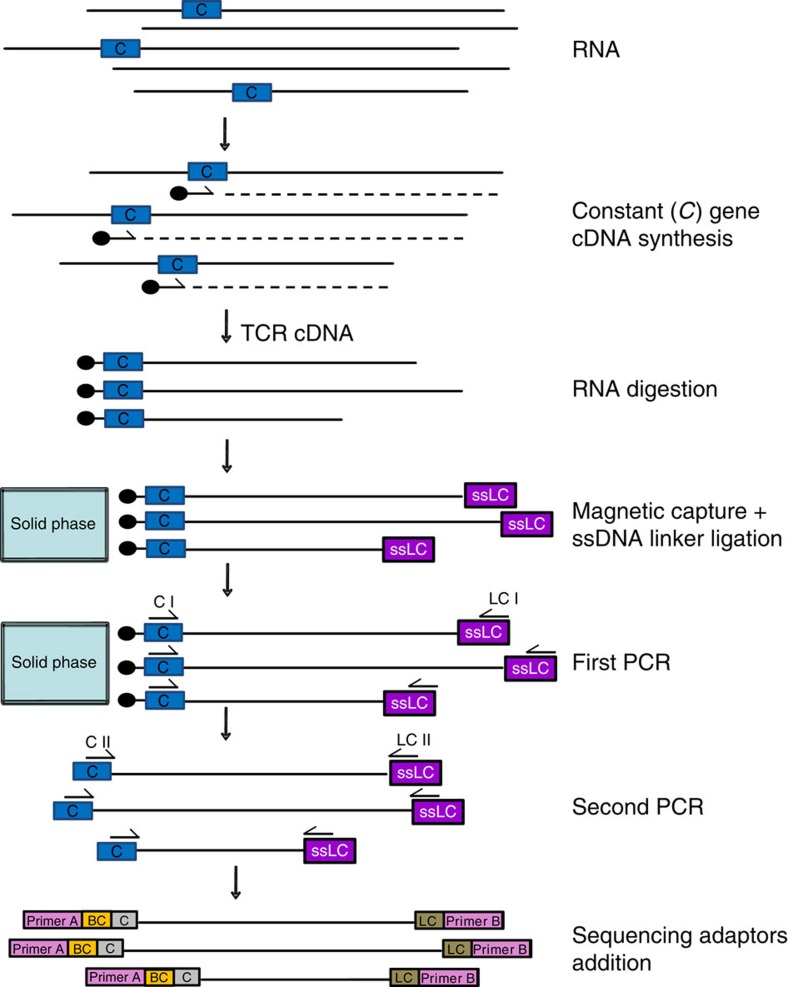
TCR-LA-MC PCR methodology. For TCR analysis, RNA was used as the starting material and gene-specific cDNA synthesis was performed using a biotinylated primer located in the constant (*C*) gene of the TCR chains. The resulting full-length cDNA fragments were magnetically captured on streptavidin beads and ligated to a known single-stranded linker cassette (ssLC). After exponential PCR amplification, sequencing adaptors were added and the PCR products were deep sequenced. *C*, constant gene; BC, sample-specific barcode.

TCR transcripts are composed of the known *C* gene and an adjacent unknown flanking region representing the rearranged *V*(*D*)*J* genes. A comparable situation, an unknown sequence adjacent to a known one, is also given in the context of viral vector integration site analyses in gene therapy, where the vector edges represent the known sequence. TCR-LA-MC PCR starts with TCR-specific cDNA synthesis using a *C* gene-specific biotinylated primer. The resulting cDNA strands are immobilized by streptavidin and a single-stranded linker cassette is ligated to their 3′ end to allow subsequent exponential amplification and deep sequencing (Fig. 1).

Using peripheral blood mononuclear cells (PBMC) and sorted T-cell fractions, we first compared available TCR-sequencing methods (Supplementary Table 1a–e). We further assessed the sensitivity and applicability of our approach. The results showed that even 10 ng of cDNA still provides a reliable representation of the existing TCR repertoire diversity (Supplementary Fig. 1a–d). Furthermore, TCR-LA-MC PCR allows T-cell identification with a resolution capacity of at least 1: 10,000 and down to the single-cell level (Supplementary Table 2a,b) and readily identifies even rare cells such as invariant natural killer T (iNKT) cells and mucosal associated invariant T-cells^15^ (Supplementary Table 2c).

### Dissection of the TCR repertoire in healthy individuals

To visualize the TCR diversity in HDs with TCR-LA-MC PCR, we first analysed the TCR repertoire in six HDs. The 454 sequencing platform was used for HDs 1, 2 and 3 and the MiSeq platform for HDs 4, 5 and 6 (Supplementary Table 3). TCR-LA-MC PCR results were consistent with even highly variable sequence numbers, ranging from hundreds to hundreds of thousands (Supplementary Fig. 2a–d). TCR-LA-MC PCR sequencing allowed a detailed, unbiased representation of functional TCR gene families, pseudogenes and open reading frame regions^16^ (Supplementary Table 4). Preferential usage of particular α- and β-chain genes was similar between all HDs (Supplementary Fig. 3a–d). A highly efficient retrieval of both α- and β-chain rearrangements of all donors was obtained, revealing the existence of nonrandom, over- as well as underrepresented, *V*–*J* pairings (Supplementary Fig. 4a,b). Preferred CDR3 lengths were observed in both chains (Supplementary Fig. 5a,b). Interestingly, we found that a similar ‘G (guanine)' preponderance, known for the G-rich composition of the *D* gene^17^,^18^ in β-chain rearrangements, is also present in the centre of α-chain rearrangements, whose recombination does not involve a *D* gene (Supplementary Fig. 5c,d).

The presence of convergence is a strong indicator of positive selection pressure on particular TCR specificities. Convergent recombination describes the phenomenon that identical TCR specificities at the amino acid level can be obtained through different DNA codes or alternative molecular routes of recombination^19^,^20^,^21^. We observed that up to 15.8% of the α-chain and up to 17.4% of the β-chain sequences in our data set encoded antigen specificities that were generated by convergent recombination (Supplementary Table 5). The importance of functional selection becomes exceedingly obvious in identical CDR3 amino acid clonotypes shared between different individuals, termed public clones^19^,^22^. Our results on human leukocyte antigen (HLA)-independent individuals (Supplementary Table 6) showed that most inter-individually shared TCR amino acid sequences were encoded by different nucleotide sequences. In HDs 1–3 we identified 626 and 272 public amino acid sequences for the α- and the β-chains, respectively. We estimated the public clone frequency for our HDs 1–6, notably HLA-independent individuals, with not more than 5.8% α- and 2.2% β-chain nucleotide clonotypes in each donor (Supplementary Fig. 6a–d). Furthermore, we screened our complete HD TCR sequence data set to identify known public clones and found TCR sequences reactive against influenza, CMV and Epstein–Barr virus^8^,^23^,^24^,^25^,^26^,^27^ (Supplementary Table 7).

### TCR repertoire in an *in vitro* model of CMV infection

CMV infection persists for life in the lymphatic tissue in 60–80% of the population. CMV is mainly controlled by CD4 and CD8 T cells^28^ directed against viral phosphoprotein 65 (pp65) and immediate early protein-1 (IE-1) (refs 28, 29, 30, 31).

**Figure 2:**
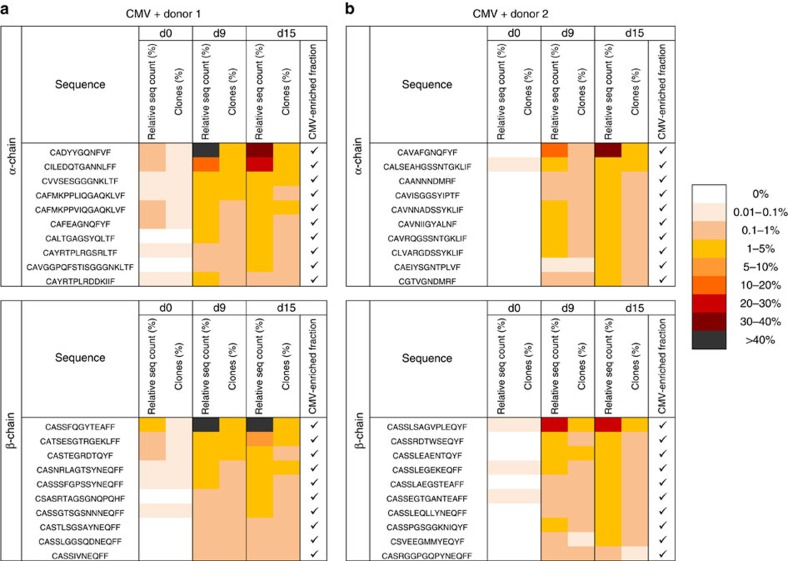
Immunomonitoring after CMV stimulation. TCR-LA-MC PCR was performed on RNA samples for two CMV-seropositive donors ((**a**) donor 1; (**b**) donor 2; upper panel, α-chain; lower panel, β-chain) at different time points after CMV stimulation. At day 15, an additional enrichment of the IFNγ-secreting fraction was performed. The 10 most predominant CMV-reactive TCR sequences identified at day 15 and their relative contribution to the total repertoire (sequence count) are shown. Retrieval frequency is indicated with a colour code ranging from light pink (0.01–0.1%) to black (>40%). We observed enrichment of defined TCR specificities over time. The number of T-cell clones (shown as %) contributing to the generation of those specificities also increased, indicating that a portion of the T-cell population is committed to the generation of specific TCR molecules. The CMV specificity of the analysed sequences was further confirmed by analysis of the enriched fraction. Two replicates were performed for day 0, 9 and 15 of each donor and sequencing results were combined. ✓, sequence was identified in the CMV enriched fraction; aa, amino acid; CDR3, complementarity-determining region 3; d, day; IFN, interferon; Seq, sequence.

To model vaccination and to study specifically activated TCR clones over time, we stimulated T-cells from two healthy CMV-seropositive donors *in vitro* with a pool of 15-mer CMV antigenic peptides spanning the full-length pp65 and IE-1 protein sequences. After stimulation, cells from both donors showed an increasing amount of IFNγ-secretion in the CD8 fraction for donor 1 and in the CD4 fraction for donor 2 (Supplementary Table 8). TCR-LA-MC PCR deep sequencing showed a polyclonal TCR repertoire at day 0 and a substantially increased contribution of CMV-reactive sequences to the TCR repertoire at day 9 after the first stimulation (Fig. 2). The intensity of convergence strongly increased after stimulation, pointing to a selection of specificities in both TCR chains. Between 48 and 169 CMV-reactive CDR3 amino acid sequences were found. These sequences showed an increasing or stable contribution over time to the total repertoire. Reactive TCR clonotypes present at day 15 could be readily backtracked on day 0 and day 9. Notably, between 0.1 and 0.4% of the T-cell clones at day 0 turned out to be reactive (Supplementary Fig. 7a–d). These changes entailed a decrease in the diversity of the TCR repertoire (Supplementary Fig. 7e,f) and the preferential usage of individual *V* and *J* genes (Supplementary Fig. 7g,h). Statistical calculation showed that even the presence of three identical amino acid is unlikely by chance alone even in 6-mer sequences (*P* value <0.05%; see Methods), demonstrating functional selection after CMV stimulation of specific TCR clones. We also identified published public clones specific for CMV infection in our two CMV-seropositive donors (Supplementary Table 9).

### TCR repertoire characterization in Sézary syndrome

**Figure 3:**
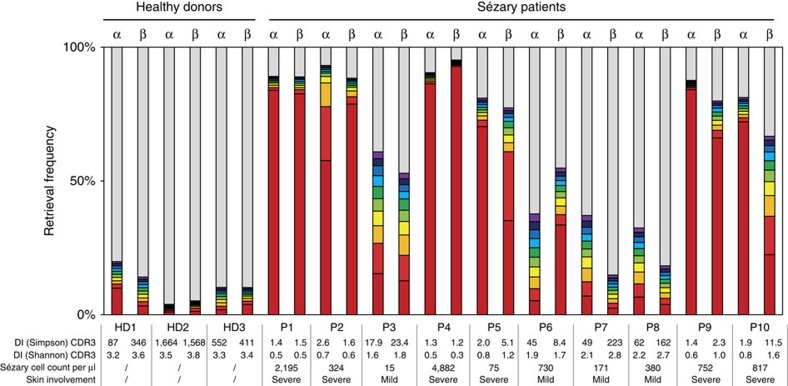
Contributions of CDR3 clonotypes in Sézary patients. The figure shows the contributions of the CDR3 aa sequences to the αβ TCR repertoire in HDs and in 10 Sézary patients. Each bar represents an individual CDR3 aa clonotype, with red and violet indicating the first and tenth most predominant sequences, respectively. Grey bars indicate the contribution of the remaining sequences identified in the analysed sample. TCR-LA-MC PCR results show that the stage of the disease correlated well with the observed TCR clonality. These results were confirmed by calculating *SI* and *SA* diversity indices (DI). The higher is the *SA* or the *SI*, the more diverse is the population. Two replicates were performed for each TCR chain of each patient and sequencing results were combined. aa, amino acid; CDR3, complementarity-determining region 3; HD, healthy donor; P, patient; *SA*, Shannon index; *SI*, Simpson index.

Repertoire information is even more relevant in pathological distortions of immunity. The pathogenesis of Sézary syndrome, an aggressive form of cutaneous T-cell lymphoma characterized by a significant number of malignant CD4 T cells circulating in the peripheral blood^32^, is still unknown and a lack of reliable clinical markers complicates clear diagnosis of the disease. We reasoned that TCR diversity analysis might readily define the extent of the pathologically distorted T-cell repertoire in this disease^33^ in a more simple and objective manner. TCR-LA-MC PCR was performed on PBMC from 10 Sézary patients at different disease stages, allowing unequivocal quantification of the degree of repertoire distortion in these patients (Fig. 3; Supplementary Table 10). Contributions of CDR3 clonotypes showed an oligoclonally distorted α- and β-chain repertoire with the emergence of a single predominant clone in patients 1, 2, 4, 5, 9 and 10, who were characterized by severe skin involvement. Patients 3, 6, 7 and 8, with mild skin involvement, showed a less-restricted clonal repertoire. Shannon (*SA*) and Simpson (*SI*) diversity indices of CDR3 amino acid for patients with a mild skin involvement indicated a less-reduced TCR repertoire, but still substantially different from a healthy situation. The clonal CDR3 sequence was different in every patient sample analysed. In several cases, the clonal *V* and *J* genes were found to be identical (Supplementary Table 11) and, interestingly, they are not commonly used in healthy individuals' TCR.

## Discussion

TCR-LA-MC PCR provides a detailed sequencing analysis of the clonal TCR repertoire. Cells, including bulk populations as well as sorted fractions (Supplementary Fig. 8a–f), blood and tissues^34^ from humans as well as from *in vivo* (Supplementary Table 12a,b, Supplementary Fig. 9a–j) and *in vitro* models can be analysed. Detailed, non-invasive monitoring of immune reactions can be obtained, enabling the discrimination of uniform and skewed TCR repertoires and its classification, even when only limiting amounts of material are available. TCR-LA-MC PCR is suitable for the identification of specific TCR clonotypes that initiate, maintain and progress infectious as well as acquired diseases.

## Methods

### Samples

To show the reproducibility of the results concerning the status of the immune repertoire constitution in different immunological conditions, we have performed TCR repertoire analysis on at least two biological human replicates. Samples from 6 healthy donors, 2 CMV-seropositive donors and 10 Sezary patients have been included in our analyses. We obtained buffy coats (∼40 ml) from healthy unrelated donors and CMV healthy seropositive individuals from the blood bank in Heidelberg and peripheral blood from 10 Sézary patients through the University Medical Center Mannheim. Each sample was obtained after informed consent of the donors. All the experiments with patient material are covered by the Medical Ethics Committee II of the Medical Faculty Mannheim of the Ruprecht-Karls-Universität Heidelberg. For the Sézary patients samples, a blinded analysis was performed: disease stage of the patients was assessed according to our TCR repertoire analysis results, then afterwards these results were compared with the clinical data/status of the patients.

After Ficoll Histopaque gradient, we cryopreserved the isolated PBMC.

### Cell line

Jurkat E6.1 cells were used for the spike-in experiments. Cells were obtained from the American Type Culture Collection (ATCC) company (ATCC, TIB-152) and tested with a multiplex cell contamination test (performed in Heidelberg by the Multiplexion company), which was conducted for the following contaminations: mycoplasma, squirrel monkey retrovirus, human-, *Macaca cynomolgus*-, mouse-, rat-, Chinese hamster-, Syrian hamster-, feline-, canine-, rabbit-, guinea pig- and *Drosophila* cells. DNA quality was determined by an internal DNA quality control. Positive and negative controls were included to monitor PCR performance. Results have shown purity of the cell line and absence of contamination.

### Mice

Spleen was taken from a 10- to 14-week-old female C57Bl/6J mouse (Jackson Laboratory, Bar Harbor, ME) raised and housed at a specific pathogen-free animal facility (DKFZ, Heidelberg) according to all applicable laws and regulations following approval by the responsible animal care (Regierungspräsidium Karlsruhe) and ethical committee.

### Single-cell sorting

We stained PBMC with fluorescein isothiocyanate (FITC)-labelled antibody to CD3 (Beckton Dickinson, Clone HIT3a, Catalogue Number 555339, 1:100) for 30 min at room temperature. We sorted single cells with a BD FACS Aria cell sorter in a 96-well plate with a V-bottom containing 6.4 μl of water and 1.6 μl of reverse transcription buffer (Life Technologies).

### RNA isolation and cDNA synthesis

We isolated RNA from the PBMC of HDs as well as CMV-seropositive donors with the RNeasy Mini (or Micro) kit (Qiagen). For iNKT cells the Arcturus PicoPure RNA Isolation kit (Life Technologies) was used. For the Sézary patients we used the RiboPure blood kit (Life Technologies) to isolate RNA from whole blood. For RNA isolation from murine spleen, the organ was first homogenized using a tissue homogenizer (Qiagen) and then processed with the RNeasy Mini kit (Qiagen). Afterwards, we performed DNA digestion with the Turbo DNA-free kit (Life Technologies). We carried out cDNA synthesis following the manufacturer's instructions using the Superscript II enzyme (Life Technologies) and biotinylated gene-specific primers for the constant gene of the TCR (Supplementary Data 1).

After cDNA synthesis, we carried out RNase H (Life Technologies) treatment and purified the resulting product with the QIAquick PCR purification kit (Qiagen).

### cDNA synthesis on single cells

After sorting single T-cells, we heated the 96-well plate at 65 °C for 2 min followed by 5 min on ice and performed cDNA synthesis using the Superscript II protocol.

### TCR ligation-anchored-magnetically captured PCR

Biotinylated fragments originated during the cDNA synthesis were captured on streptavidin magnetic beads (Life Technologies). For each sample, 200 μg magnetic beads were exposed for 60 s to a magnetic field, washed twice with PBS/0.1% BSA, once with 3 M LiCl and finally resuspended in 6 M LiCl (purified cDNA volume:beads volume=1:1). Samples were incubated on a shaker at 300 r.p.m. After an overnight enrichment of the biotinylated cDNA molecules, samples were washed with water to remove the unbound unspecific fragments and then resuspended in 10 μl of the ligation mastermix. We used 0.1 ng–1 μg (1 μg for healthy donors and CMV samples, 200 ng for Sézary samples, everything for the enriched CMV fraction, the spike-in experiment and for the sorted fractions, 1 μg–0.1 ng for the limiting dilution experiment) of the captured cDNA molecules as a template for the ligation procedure. We ligated a single-stranded DNA linker phosphorylated at its 5′ end and carrying a dideoxycytidine at its 3′ end (single-stranded linker cassette (ssLC)) to the unknown genomic end of the amplicons. Ligation was carried out using the CircLigase enzyme (Epicentre) at 60 °C for 1 h in a total volume of 10 μl.

At the end of the reaction, samples were washed with water, resuspended in 10 μl water and 1 μl was further processed. To amplify the CDR3 region of the TCR molecules, we performed two exponential PCR (50 μl total volume) using nested primers for the constant gene of the TCR chains and for the ssLC (Supplementary Data 1). Each primer was used at a concentration of 8.4 pmol. We used the following PCR programme: initial denaturation at 95 °C for 120 s followed by 10 cycles (95 °C for 45 s, 55 °C for 45 s and 72 °C for 20 s) and an additional denaturation at 90 °C for 120 s followed by 25 cycles (90 °C for 45 s, 55 °C for 45 s and 72 °C for 20 s). A biotinylated primer for the constant gene of the TCR molecule was used in the first exponential PCR. Between first and second exponential PCR an additional magnetic capture was performed to increase the specificity of the reaction by removing all the unwanted fragments. After washing as described above, 20 μl beads in 6 M LiCl were mixed with 20 μl of PCR product (1:1 ratio) and the samples were incubated for at least 1 h on a shaker at 300 r.p.m. At the end of the incubation, samples were washed with water to remove the unspecific fragments that have not been bound to the beads, resuspended in 10 μl 0.1 N NaOH and incubated 10 min on a shaker at 300 r.p.m. Denaturation with NaOH was performed to obtain the non-biotinylated strands. A volume of 2 μl of the denatured product was used for the second exponential PCR. We used the same PCR programme of the first exponential PCR with the addition of a final extension step at 72 °C for 120 s. Afterwards, we purified the PCR amplicons using AmPure beads (Beckman Coulter) and prepared them for sequencing. Repetitive TCR-LA-MC PCR analyses were performed for healthy donors, CMV donors, Sézary patients and for the spike-in experiment.

### High-throughput sequencing of TCR-LA-MC PCR amplicons

We used the 454 GS Flx Titanium Platform for long amplicon sequencing. To add specific GS Titanium adaptors (A and B) to both ends of the PCR amplicons, we performed Fusion primer PCR. To allow simultaneous analysis of multiple samples, we incorporated barcodes of 6 or 10 bp into the fusion primer A-TCR. We amplified 40 ng of purified PCR products with the following programme: initial denaturation for 120 s at 95 °C, followed by 12 cycles (95 °C for 45 s, 60 °C for 45 s and 72 °C for 60 s) and final elongation for 300 s at 72 °C. We purified PCR amplicons with AmPure beads (Beckman Coulter) and sequenced them. With recent improvements in the Illumina sequencing technology, the sequencing of long fragments (>250 bp from each side) has become possible allowing detection of the CDR3 region without the need to fragment the sequences. Thus, the MiSeq platform was used for the sequencing of iNKT cells, T-cell subsets, HDs 4, 5 and 6 and limiting dilution on Sézary samples. As described for sequencing with the 454 platform, a Fusion primer PCR was performed with 50 ng of purified amplicon product and special fusion primers harbouring Illumina MiSeq sequencing adaptors. A double barcoded strategy was used allowing the tagging of individual samples with a barcoded (12 bp) linker cassette in addition to the barcoded fusion primer. Fusion primers used are listed in Supplementary Data 1. Each primer was used at a concentration of 5 pmol.

### Comparison RACE PCR and TCR-LA-MC PCR

Different methodologies have been tested for the sequencing of the TCR repertoire. We have performed several variants of a multiplex PCR^35^ approach both on DNA and RNA (MP1, Multiplex PCR; MP2, Multiplex PCR followed by a nested Multiplex PCR; MP3, Multiplex PCR followed by a nested Multiplex PCR (reactions performed with the Pfx polymerase); MP4, Multiplex PCR with biotinylated *J* primers followed by magnetic capture and by a nested Multiplex PCR; MP5, Multiplex PCR followed by Microcon30 treatment and by a nested Multiplex PCR; MP6, Linear amplification with biotinylated *J* primers followed by a Multiplex PCR with biotinylated primers, magnetic capture and a nested Multiplex PCR), a commercially available kit (TCR Express Human TCR Vβ Repertoire Clonality Detecting System (BioMed Immunotech)) on RNA and different variants^2^,^5^ of RACE PCR on RNA. In the case of the different variants of the multiplex PCR and for the amplicons derived from the TCR Express Kit, we added by ligation two double-stranded fragments harbouring known sequences to the PCR amplicons. Sequencing adaptors were included by Fusion Primer PCR. RACE PCR^2^ amplicons were prepared for sequencing as described for the TCR-LA-MC PCR.

As the focus of the paper is the use of PCR-based approaches devoid of multiplexing primers for the TCR sequencing of expressed molecules, after an initial test we have concentrated our work on TCR-LA-MC PCR and a recently published adaptation of the conventional RACE PCR^5^.

Three experiments were performed to compare RACE PCR^5^ and TCR-LA-MC PCR efficiency as described in Supplementary Table 1b–e. RNA has been isolated from PBMC of two healthy donors starting from 3 × 10^7^ cells for donor 1 (labelled as PBMC1) and 5 × 10^7^ for donor 2 (labelled as PBMC2). A unit of 18 μg of RNA was retrieved for PBMC1 and 12 μg for PBMC2. A unit of 150 ng RNA was used for each sample condition. In the first experiment, TCR-LA-MC PCR has been performed either by using Superscipt II (Life Technologies) as described in the manuscript or SmartScribe (Clontech) reverse transcriptase. SmartScribe reverse transcriptase (Clontech) has been used for cDNA synthesis following the manufacturer's instruction. In the second experiment, the influence of magnetic capture introduction in the RACE PCR protocol has been evaluated. In the third experiment, primers used in the RACE PCR protocol have been introduced in the TCR-LA-MC PCR procedure.

The respective PCR (50 μl total volume) was prepared with 2 × Pfx amplification buffer, 0.3 mM dNTP, 10 pmol of each primer and 1 unit of Platinum Pfx DNA polymerase (Life Technologies). First PCR amplification was carried out as follows: initial denaturation at 94 °C for 2 min, 18 cycles of 94 °C for 30 s, 55 °C for 30 s, 68 °C for 45 s and a final elongation at 68 °C for 5 min. For the second exponential PCR 27 cycles were used for both methodologies. Sequencing adaptors were directly added in the second PCR and amplicons were purified using the AmPure beads (Beckman Coulter). Samples were sequenced with MiSeq paired-end 250 bp and the MiTCR software^36^ was used for the analysis of the sequencing results.

RACE PCR protocol was performed as described in Bolotin *et al*.^5^ First-strand cDNA synthesis was performed using the SmartScribe enzyme in the presence of a constant gene-specific primer and of a template-switching primer added during the reaction. For the first and second exponential PCR, 1 μl undiluted first-strand cDNA and 1 μl first PCR product were used as input material, respectively. A volume of 15 pmol of each primer was used in the first exponential PCR. Second exponential PCR primers have been generated by fusing the adaptor sequence required for the Illumina platform with the MmeStep1 and MmeSmart20 primers^5^. A volume of 7.5 pmol of MiSMmeStep1 and 0.75 pmol of MiSMmeSmart20 were used. TCR-specific primers were prepared accordingly as described in the main text for the TCR-LA-MC PCR and used at a concentration of 7.5 pmol.

When magnetic capture was introduced in the RACE PCR protocol to capture TCR-specific cDNA fragments, a biotinylated BC1R *C* gene-specific primer was used during the reverse transcription and afterwards TCR-specific cDNA molecules were captured with 200 μg pre-washed streptavidin magnetic beads (cDNA volume:beads volume=1:1). Samples were incubated overnight on a shaker at 300 r.p.m. The day after, samples were washed and resuspended in 10 μl water. A volume of 1 μl was then used for the first exponential PCR. When magnetic capture was performed both after cDNA and first exponential PCR or only after the first expo, a biotinylated *C* gene-specific BC2R primer was used in the PCR. Then 20 μl of the biotinlyted PCR products were captured with 200 μg pre-washed streptavidin magnetic beads and incubated for at least 1 h on a shaker at 300 r.p.m. Samples were washed with water to remove the unspecific fragments that have not been bound to the beads, resuspended in 10 μl 0.1 N NaOH and incubated 10 min on a shaker at 300 r.p.m. Then 1 μl of the denatured product was used for the second exponential PCR.

TCR-LA-MC PCR was performed as described in the manuscript with the following differences: (1) cDNA synthesis was perfomed using either Superscript II or Smartscribe reverse transcriptase; (2) between first and second exponential PCR no magnetic capture was performed; instead 1 μl of first PCR product was directly used as input for the second exponential PCR; and (3) adaptor sequences were directly added in the second exponential PCR.

In addition, when RACE PCR primers were used in the TCR-LA-MC PCR protocol, BC1R biotinylated primer was used for the cDNA synthesis and BC2R biotinylated primer was used in the first exponential PCR. Furthermore, a different linker cassette was ligated during the single-stranded ligation (PHO-LC2-DDC) allowing then the usage of the RACE primers M1 in the first exponential PCR and MiSMmeStep1 as well as MiSMmeSmart20 in the second exponential PCR.

### Antigens

We used a pool of 15-mer peptides with 11 amino acid overlap spanning the full CMV pp65 and IE-1 proteins (Peptivator, Miltenyi Biotec).

### Cell stimulations with antigenic peptides

We thawed cryopreserved PBMC and incubated them overnight in RPMI (Gibco) supplemented with 5% human serum (Sigma-Aldrich), 100 U ml^−1^ penicillin/streptomycin (Lonza) and 2 mM L-glutamine (Lonza).

To measure the number of CMV-specific T cells in the isolated PBMC, we stimulated cells with a final concentration of 1 μg each peptide per ml. After 6 h of incubation at 37 °C and 5% CO_2_, we diluted the cells to a concentration of 2 million per ml and added 30 U ml^−1^ human recombinant IL-2. We refreshed the cell medium and IL-2 every 2–3 days and added IL-7 (10 ng ml^−1^) once a week. After 9 days of culture, we performed restimulation of the CMV-specific cells. We loaded autologous irradiated (50 Gy) PBMC for 4 h with the peptide pools and used them as feeder cells (stimulator:effector ratio of 1:3). We kept the cells in culture for additional 6 days. At days 0, 9 and 15, we used an aliquot of cells for IFNγ FACS staining and an additional aliquot for TCR repertoire analysis.

### Flow cytometric analysis

We performed phenotypic characterization of the stimulated PBMC by using directly conjugated antibodies (Beckton Dickinson). After 2 h of incubation of the PBMC with the peptide pools, we added Brefeldin A (BFA) at a concentration of 7.5 μg ml^−1^ and incubated the cells for additional 4 h. We washed the cells with PBS supplemented with 0.1% BSA (w/v) containing 1 μg ml^−1^ BFA (PBS/BSA/BFA) and stained them with an Alexa 700-labelled antibody to CD4 (Beckton Dickinson, Clone RPA-T4, Catalogue Number 557922, 1:50) and an allophycocyanin (APC)-labelled antibody to CD8 (Beckton Dickinson, Clone RPA-T8, Catalogue Number 555369, 1:50) in a total volume of 100 μl for 30 min at 4 °C. We then washed the cells in PBS/BSA/BFA, fixed them with PBS containing 4% (w/v) paraformaldehyde for 20 min at room temperature and permeabilized them with PBS with 1% BSA (w/v) and 0.1% saponin (w/v) (Sigma-Aldrich) (perm buffer) at room temperature for 10 min. Afterwards, we washed the cells and incubated them for intracellular staining with a FITC-labelled antibody to IFNγ (Beckton Dickinson, Clone B27, Catalogue Number 554700, 1:50) in 100 μl of perm buffer for 30 min at room temperature. We acquired fluorescent events with a LSRII flow cytometer (Beckton Dickinson) and performed lymphocyte gating to determine the T-cell population. Sorting of T-cell subsets was performed using directly conjugated antibodies (Beckton Dickinson) for membrane staining of CD3 (Beckton Dickinson, Clone HIT3a, Catalogue Number 555339, 1:100), CD4 (Beckton Dickinson, Clone RPA-T4, Catalogue Number 557922, 1:50), CD8 (Beckton Dickinson, Clone RPA-T8, Catalogue Number 555369, 1:50) and CD45RO (Beckton Dickinson, Clone UCHL1, Catalogue Number 555493, 1:100) molecules. The stained cells were then sorted using the BD FACS Aria (Beckton Dickinson).

### Enrichment of IFNγ-secreting cells

To isolate IFNγ-secreting cells, we stimulated the cells with CMV pp65 and IE-1 pools for 4 h on day 15 as previously described. We then collected the cells, washed them with PBS and performed staining and enrichment with the IFNγ capture assay (Miltenyi Biotec). We first added an IFNγ-catch reagent to label the cells, incubated them at 37 °C for 45 min and then stained them with a phycoerythrin (PE)-labelled antibody to the IFNγ. We enriched for IFNγ-secreting cells by using anti-PE microbeads and separated them using the MACS system (Miltenyi Biotec). We stored the isolated cells for TCR repertoire analysis.

### Isolation of iNKT cells

iNKT cells represent a very rare population of T cells, being present at a frequency ranging between 0.01 and 1% (ref. 15). First, we performed magnetic separation using anti-iNKT microbeads (Miltenyi Biotec) followed by staining of the labelled cells. A total of 50 million frozen PBMC was used as starting material. After centrifugation, the cells were resuspended in 400 μl of cold buffer solution (PBS, pH 7.2, 0.5% BSA, 2 mM EDTA) and 100 μl anti-iNKT microbeads were added. Samples were incubated for 15 min in the fridge. At the end of the incubation time, 50 μl of Anti-iNKT-PE antibody (Miltenyi Biotec, Clone 6B11, Catalogue Number 130–098–128) were used for the staining and the cells were kept in the fridge for 5 min. After a washing step, magnetic separation using a MACS separator (Miltenyi Biotec) and MACS columns (Miltenyi Biotec) was carried out. To enrich the purity of the separated fraction, an additional separation was performed over a second column and cells were stained with Fluorogold (Life Technologies) to label dead cells. Second, FACS sorting was performed, retrieving 10,215 cells.

### Bioinformatic analysis of TCR amplicons

After removal of the introduced linker cassette, we aligned the trimmed sequences via BLAT^37^ (*-stepSize*=5 -*minIdentity*=0 -*minScore*=0) to a reference set of TCR genes. We obtained these gene data sets for mouse and human from the IMGT/GeneDB database^38^. If we identified the *J* gene as well as the *V* gene for a given sequence in the amplicon via BLAT, we further analysed its CDR3 junction. We selected the terminal conserved cysteine in the *V* gene and the phenylalanine of the mainly conserved FGXG motif in the *J* gene as junction boundaries in accordance with multiple amino acid sequence alignments of corresponding alleles obtained from IMGT.

For the β-chain we identified the *D* gene within the junction nucleotides via pairwise sequence alignment^39^,^40^. We reported a *D* gene name if for the relatively short *D* sequences an alignment of 10 or more nucleotides with ≥90% sequence identity or of 3 or more nucleotides with 100% sequence identity between the reference sequence and a part of the junction could be verified. We identified and reported additional nucleotides (N nucleotides) inserted between the *D* and J genes, *V* and *D* genes or *V* and *J* genes (α-chain). The source code of our own TCR sequence analysis bioinformatical tool may be available on request.

For some experiments, analysis of the TCR-sequencing results was performed by using the MiTCR software^36^.

### Error estimation

Being aware of the errors deriving from PCR amplification and sequencing, we performed an estimation of the potential error rate by comparing the sequenced *V* gene portion of the CDR3 region with its germline sequence. We performed a sequence alignment using BLAST and carried out the calculation of the error rate by considering sequences harbouring one or more mismatches. The results showed that the error rate was ∼2% per nucleotide.

### Statistics to identify identical amino acids in a sequence

Under the assumption that the amino acids are equiprobable (*p*=1/20) and independent we calculated the probability of finding subsequences of *m* amino acids in sequences of length *k* using the following formula


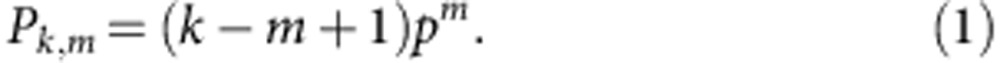


derived from the elementary rules of probability.

### Heat-map

We used log-odd scores as non trivial representations of the abundance of *V*–*J* pairs. Log-odds can be written as *l*_*V*,*J*_*=*log_2_(*p*_*V*,*J*_*/p*_*V*_*p*_*J*_)*. p*_*V*,*J*_ is the frequency that gene *V* is fused with gene *J* and *p*_*V*_ and *p*_*J*_ are the background uniform probability with which the *V* and *J* genes occur in the samples. We expressed the resulting *l*_*V*,*J*_ score in bit due to the logarithm base.

### CMV profile selection

From the total sequences retrieved, we selected the amino acid sequences consistent with an expansion profile. We defined as expansion a profile where the contribution of a sequence *s* between day 0 and day 9 is increasing and between day 9 and day 15 is at least stable. The latter condition was due to a saturation phenomenon that could affect the frequencies of strongly activated species. We computed the distribution of the negative differences between day 15 and day 9 and then considered all those species where this difference lays under the 25th percentile as stable.

### Diversity indices

To estimate the diversity in the TCR repertoire, we calculated two different diversity indices: Shannon index (*SA*)^41^ and Simpson index (*SI*)^42^. Both of them take in consideration the two components that constitute the concept of diversity, the richness of a population and its homogeneity. The richness of a population is defined by its total number of species (for example, CDR3 amino acid sequences) and is estimated with the Shannon index as follows:


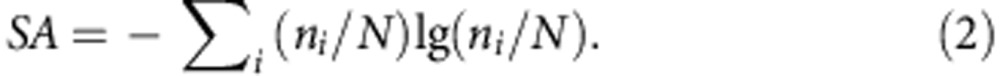


Homogeneity measures the distribution of the species (for example, CDR3 amino-acid sequences) in the population and is calculated with the Simpson index as follows:


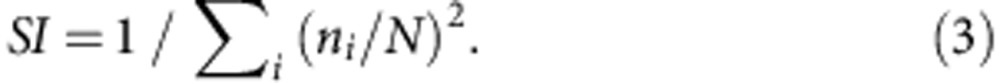


where *i* is an index that is chosen between 1 and the number of species *s*, *n*_*i*_ is the number of sequencing reads in species *i* and *N* is the total number of reads.

## Additional information

**Accession codes:** The TCR-sequencing data generated in this paper have been deposited in the SRA database under the accession code SRP059581.

**How to cite this article:** Ruggiero, E. *et al*. High-resolution analysis of the human T-cell receptor repertoire. *Nat. Commun.* 6:8081 doi: 10.1038/ncomms9081 (2015).

## Supplementary Material

Supplementary Information Supplementary Figures 1-9, Supplementary Tables 1-12 and Supplementary References

Supplementary Data 1 Oligonucleotides. Primers and oligonucleotides used for the TCR sequencing approaches are listed in the table. B, biotin; DDC, 2′,3′ dideoxycytidin; PHO, phosphorylation; LC, linker cassette; hTCR, human T-cell receptor; mTCR, murine T-cell receptor; TSW, template switching; BC, barcoded; FuP, fusion primer; barcodes are indicated as N.
