# Supplementary material for: High-resolution analysis of the human T-cell receptor repertoire
Source: Nat Commun. 2015 Sep 1;6:8081. doi: 10.1038/ncomms9081 (PMC4569693; doi:10.1038/ncomms9081)
Supplement: Supplementary Information — Supplementary Figures 1-9, Supplementary Tables 1-12 and Supplementary References [file ncomms9081-s1.pdf]

Supplementary Figure 1

a

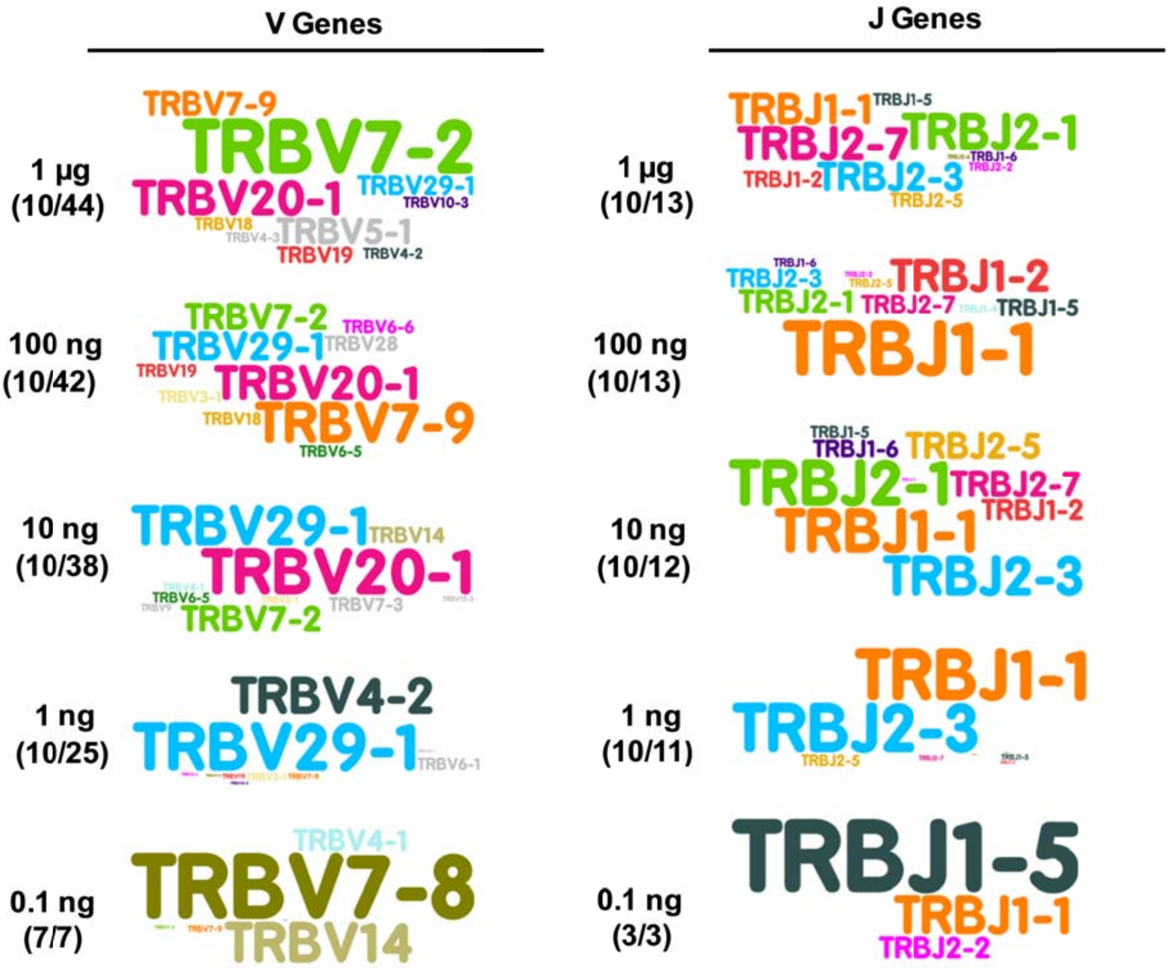

b

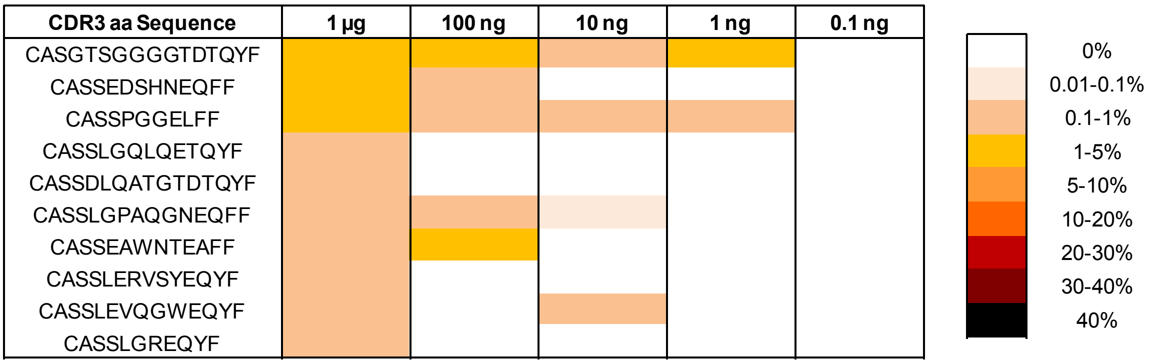

c

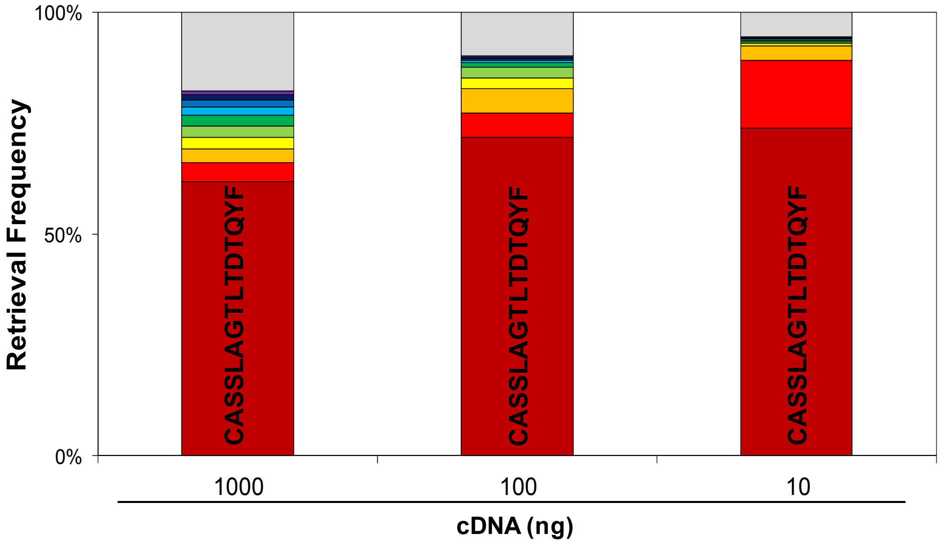
Retrieval Frequency
100%
50%
0%
CASSLAGTLTDTQYF
CASSLAGTLTDTQYF
CASSLAGTLTDTQYF
1000
100
10
cDNA (ng)

d

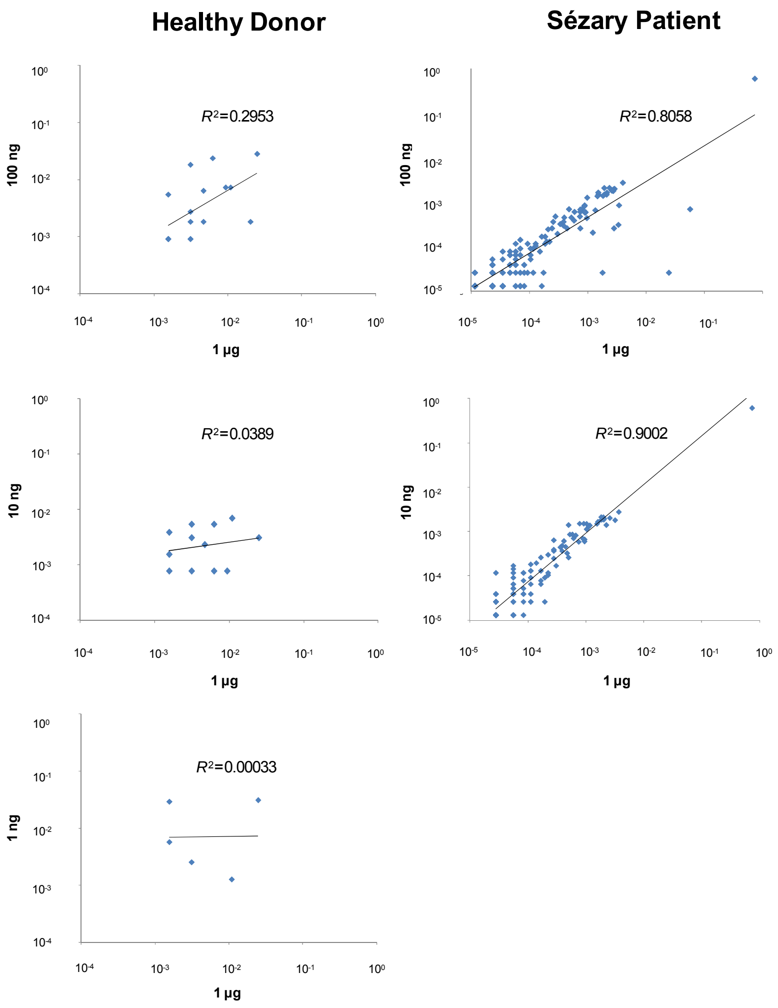
Healthy Donor
Sézary Patient
R^2=0.2953
R^2=0.8058
R^2=0.0389
R^2=0.9002
R^2=0.00033
100 ng
10 ng
1 ng
1 µg

Supplementary Figure 1. Limiting dilution experiments. Limiting dilution experiments have been performed on a HD sample (**a, b, d**) and on a sample from a Sézary syndrome patient (**c, d**). (**a**) *V* (left side) and *J* (right side) gene usage for HD samples (input cDNA range: 1,000 ng-0.1 ng) have been represented as clouds. Different letter sizes represent the gene usage as retrieval frequency (in percentage). Different colours display different genes, the same colour in different clouds represents the occurrence of the same gene in more than one dataset. Genes found in only one sample have been labeled in grey. Numbers in brackets denote "illustrated genes/detected genes". (**b**) TCR clonotypes contribution for HD samples. The heat map displays in different colours the retrieval frequency of each of the 10 most predominant TCR sequences identified by TCR-LA-MC PCR in the sample with 1 µg of input material in the following dilution series. (**c**) The 10 most predominant CDR3 aa clonotypes identified in a Sézary patient sample (input cDNA range: 1,000 ng-10 ng) are represented in the graphic. Each bar represents an individual CDR3 aa clonotype, with red and violet indicating the first and tenth most predominant sequence. Grey bars indicate the remaining sequences identified in the analysed sample. (**d**) Pearson correlation test was performed on the results of the two limiting dilution experiments. For each dataset the retrieval frequency of the CDR3 aa sequences was used to determine the Pearson correlation coefficient. Log-transformation of the data was performed. Whereas for the limiting dilution experiment on HD samples the Pearson correlation coefficient, R^2, showed a weak correlation between each 2 datasets (1 µg, 100 ng, 10 ng, 1 ng), Pearson correlation coefficients for the Sézary samples showed a nearly linear correlation between the analysed datasets (1 µg, 100 ng, 10 ng). Overall, results show that 10 ng of cDNA can provide a good representation of the TCR repertoire diversity. HD, healthy donor; TCR-LA-MC, TCR ligation anchored-magnetically captured; aa, amino acid; *V*, variable; *J*, joining; *TRBV*, TCR β-chain variable gene; *TRBJ*, TCR β-chain joining gene; CDR3, complementarity determining region 3.

Supplementary Figure 2

a

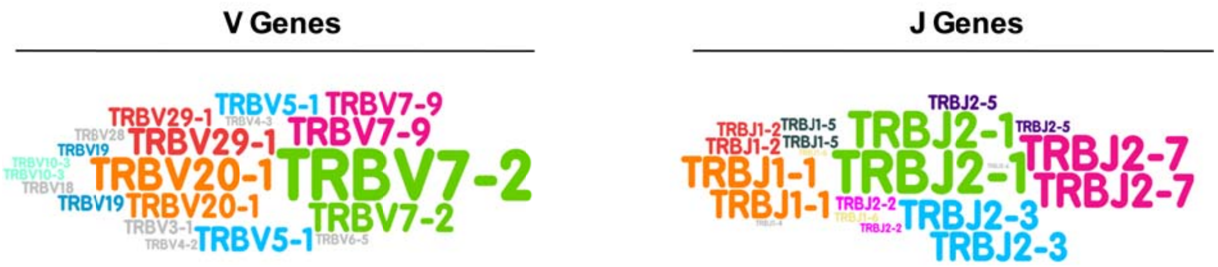

b

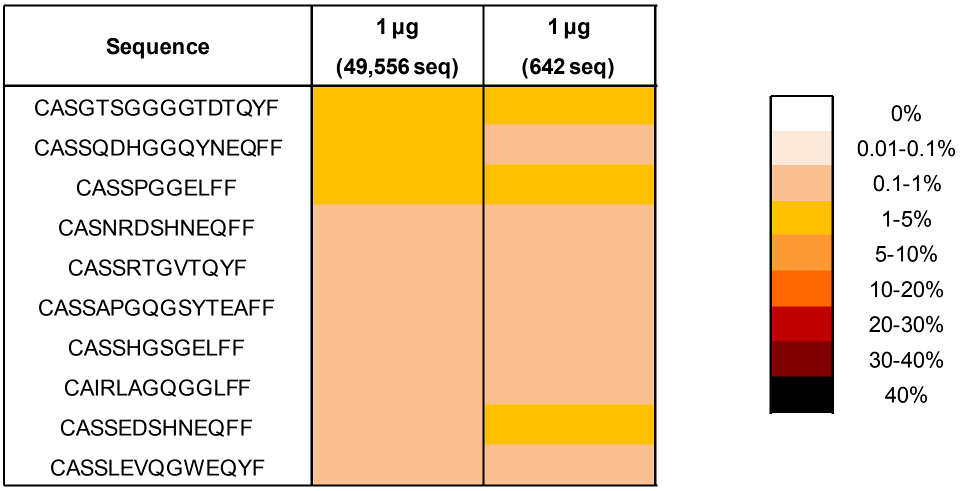

c

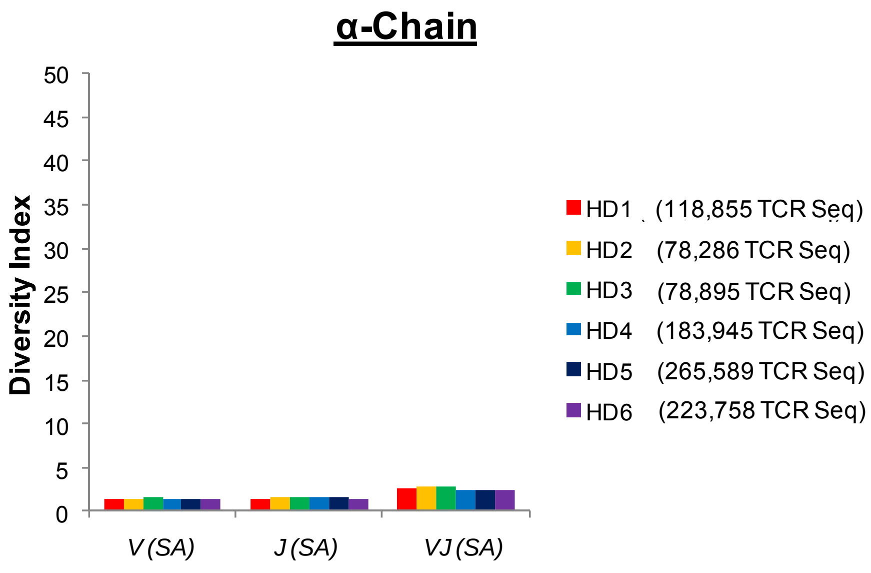

d

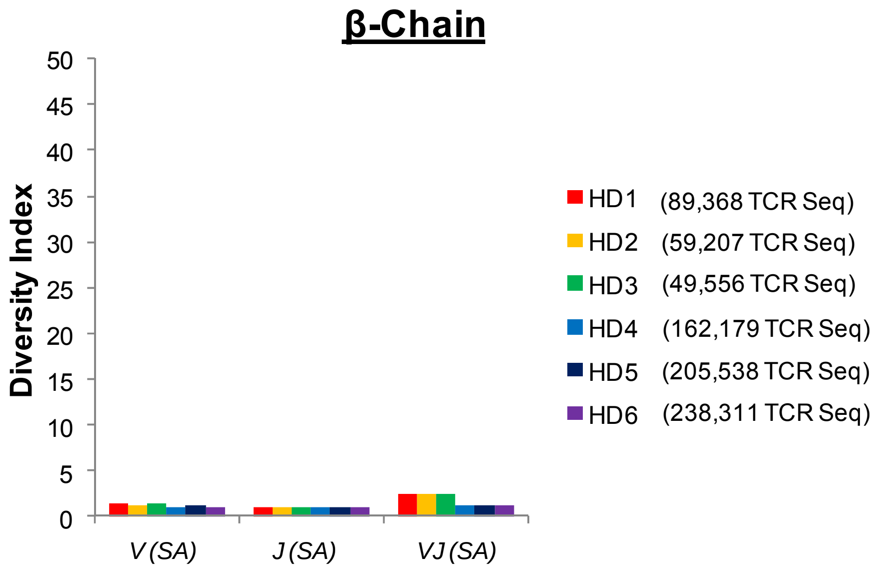

Supplementary Figure 2. Comparison of different numbers of sequencing reads. (**a, b**) TCR-LA-MC PCR sequencing has been performed on two PBMC samples (1 µg cDNA starting material) of a HD. 49,556 and 642 sequences have been retrieved and analysed to investigate whether a low amount of reads can provide a reliable overview of the TCR clonality. *V* (**a,** left side) and *J* gene (**a**, right side) usage has been represented as cloud pictures. Different colours display different genes, the same colour in a cloud represents the occurrence of the same gene in the 2 different sequence datasets. Genes found in only one sample have been labeled in grey. The size of every word reflects the retrieval frequency of a specific gene. The 10 most used *V* and the 5 most used *J* genes have been considered. Results show the same pattern of *V* and *J* gene usage in the 2 analysed datasets. (**b**) TCR aa clonotype contribution has been investigated for the 2 analysed datasets. Retrieval frequencies of the individual CDR3 aa sequences are reported in different colours: the darker the colour, the stronger is the frequency of a defined clonotype in the analysed TCR repertoire. Results show that the 10 most prominant CDR3 aa sequences identified in the dataset formed by 49,556 sequences were identified with comparable frequency contributions in the smaller dataset (642 sequences). (**c, d**) To investigate if the number of α- and β-chain sequence reads retrieved by the sequencing of the different HD samples is reliable and gives a good sampling of the TCR pool, we used Shannon diversity index (*SA*). We found a similar *SA* value for *V* genes, *J* genes and *V-J* pairings in each HD α- (**c**) and β-chain (**d**) dataset that was not affected by the sequencing technology and by the number of retrieved sequences. TCR-LA-MC PCR, TCR ligation anchored-magnetically captured PCR; HD, healthy donor; *V*, variable; *J*, joining; *TRBV*, TCR β-chain variable gene; *TRBJ*, TCR β-chain joining gene; CDR3, complementarity determining region 3; seq, sequences; TCR seq, sequences where the CDR3 region was identified; aa, amino acid.

Supplementary Figure 3

a

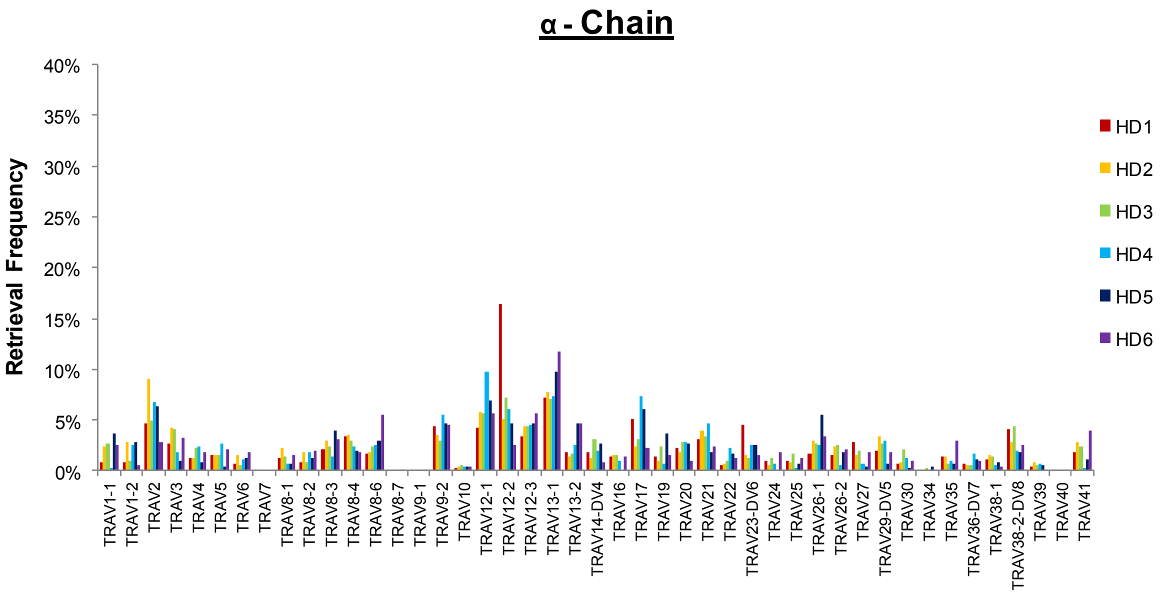

b

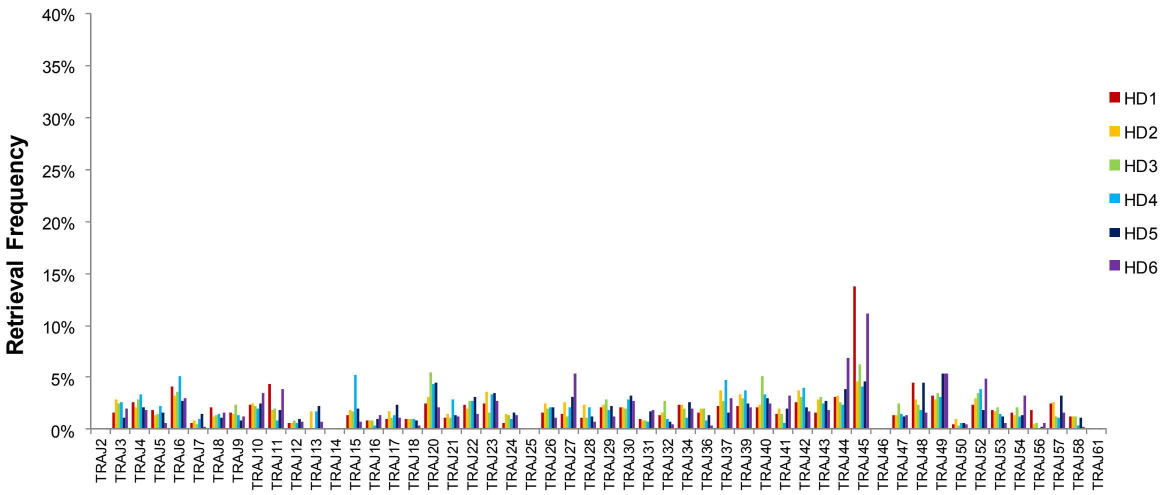

c

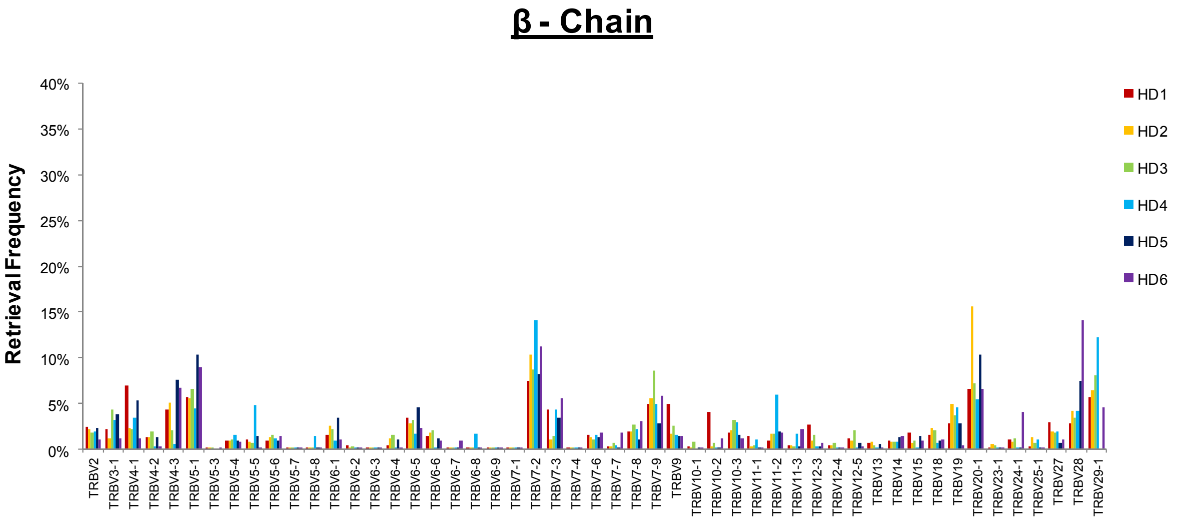

d

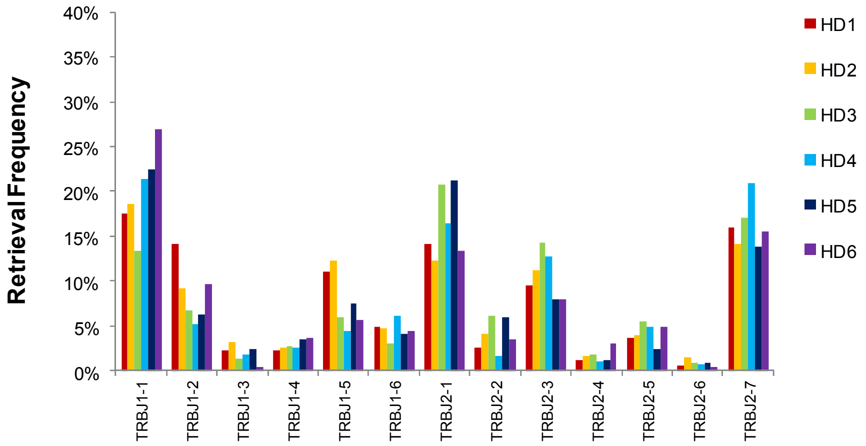

Supplementary Figure 3. *V* and *J* gene usage for alpha- and beta-chains in healthy donors. *V* (**a** and **c**) and *J* (**b** and **d**) gene usage for healthy donor 1-6 (**a, b**, α-chain; **c, d**, β-chain) are represented as histograms indicating a comparable gene usage for all 6 donors. Each donor is shown with a different colour. For each donor, two replicates have been performed and the sequencing results have been combined. *TRAV*, TCR variable gene of α-chain; *TRAJ*, TCR joining gene of α-chain; *TRBV*, TCR β-chain variable gene; *TRBJ*, TCR β-chain joining gene.

Supplementary Figure 4

a

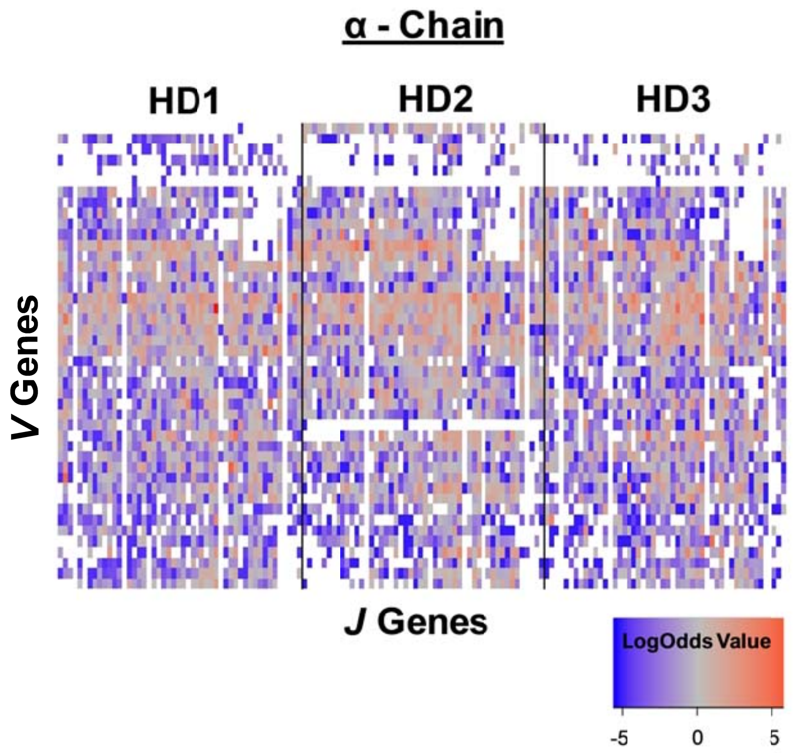

b

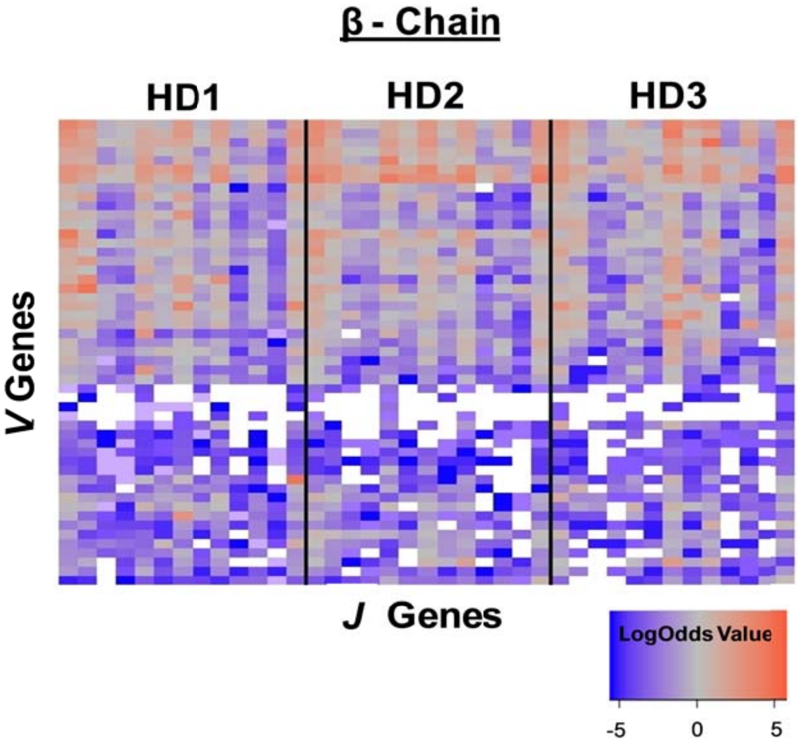

Supplementary Figure 4. *V-J* pairings for alpha- and beta-chains in healthy donors. Odd values in heatmaps (**a**, α-chain; **b**, β-chain) highlight different combinations of *V* and *J* genes with a blue and red colour code indicating underrepresented and overrepresented pairings. The same pattern of *VJ* pairing has been observed in different HD. Each coloured cell represents a defined *VJ* pairing. Results for HD 1-3 are shown in the figure. For each donor, two replicates have been performed and the sequencing results have been combined. *V*, variable, *J*, joining; HD, healthy donor.

Supplementary Figure 5

a

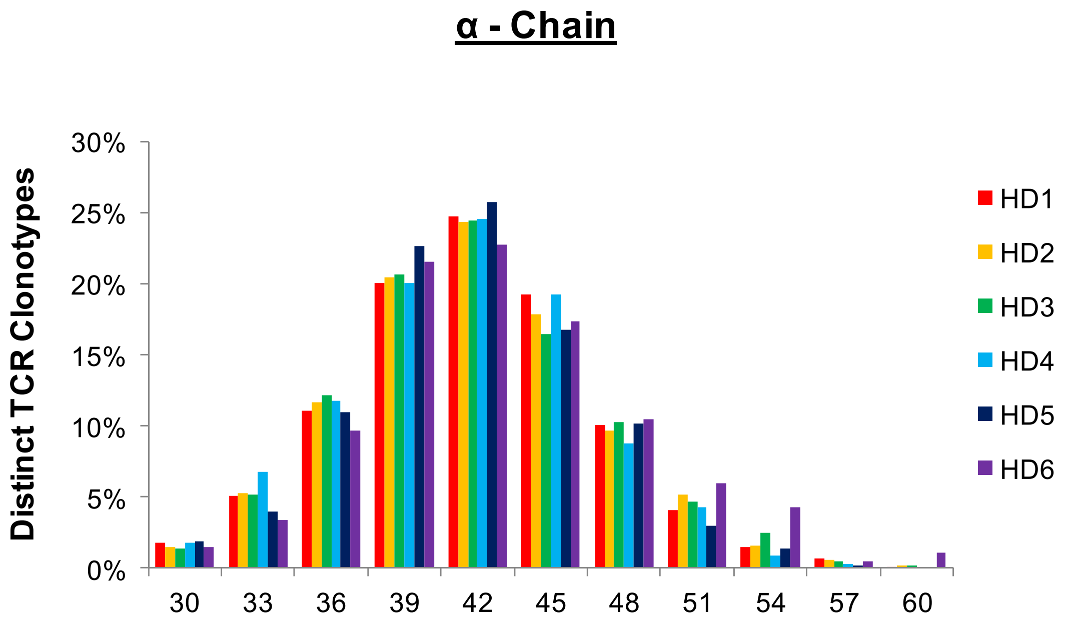

b

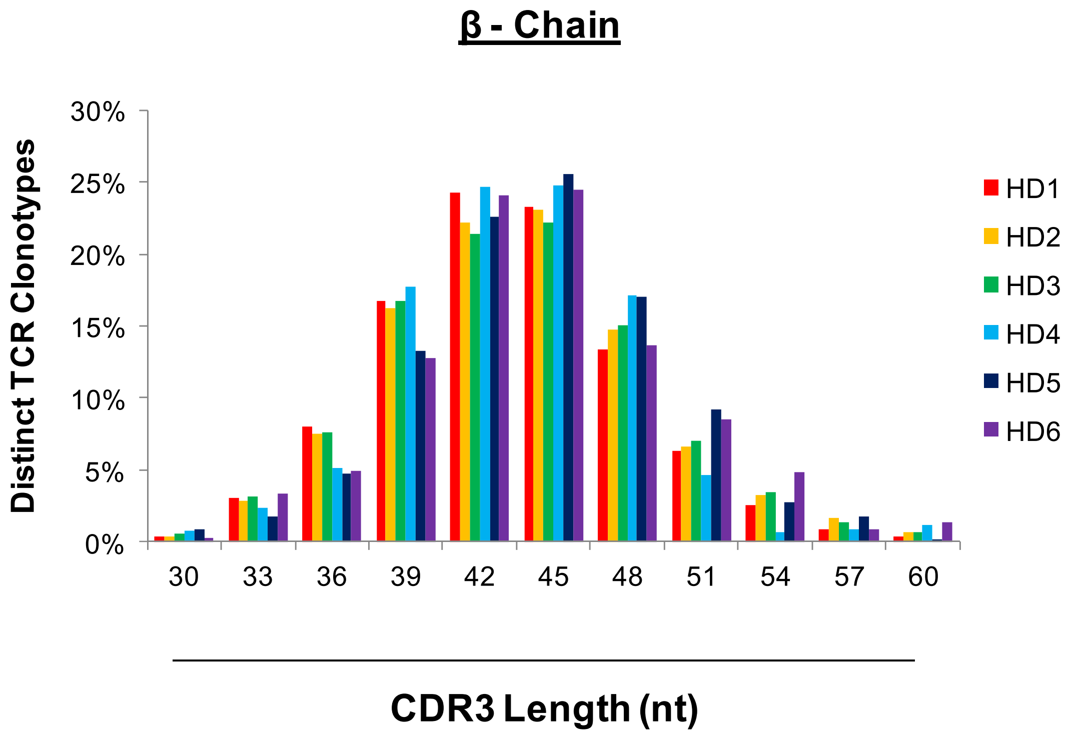

c

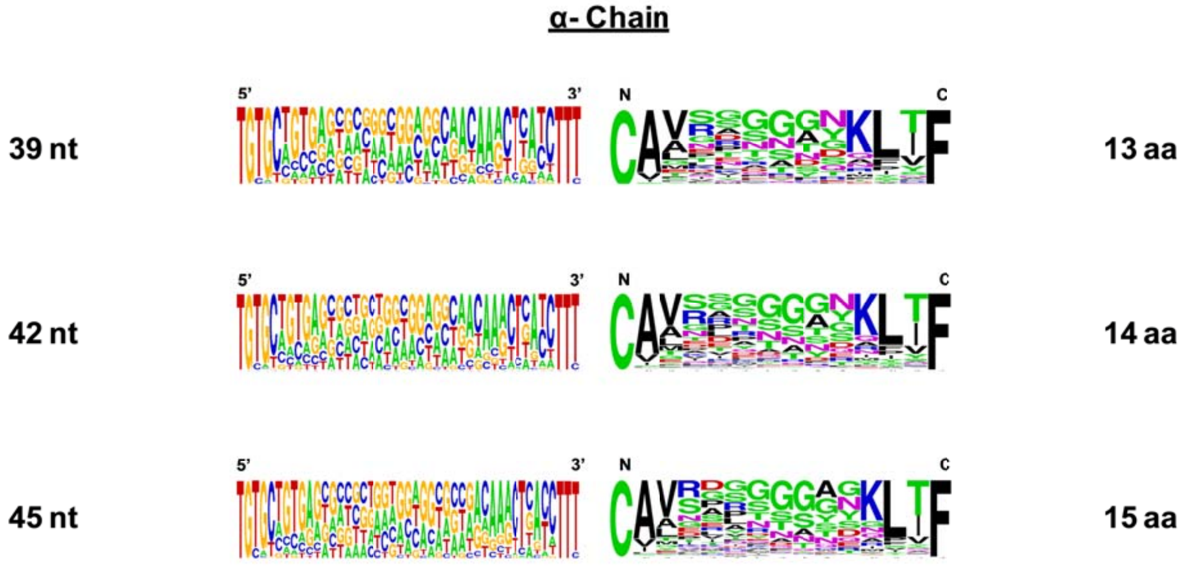

d

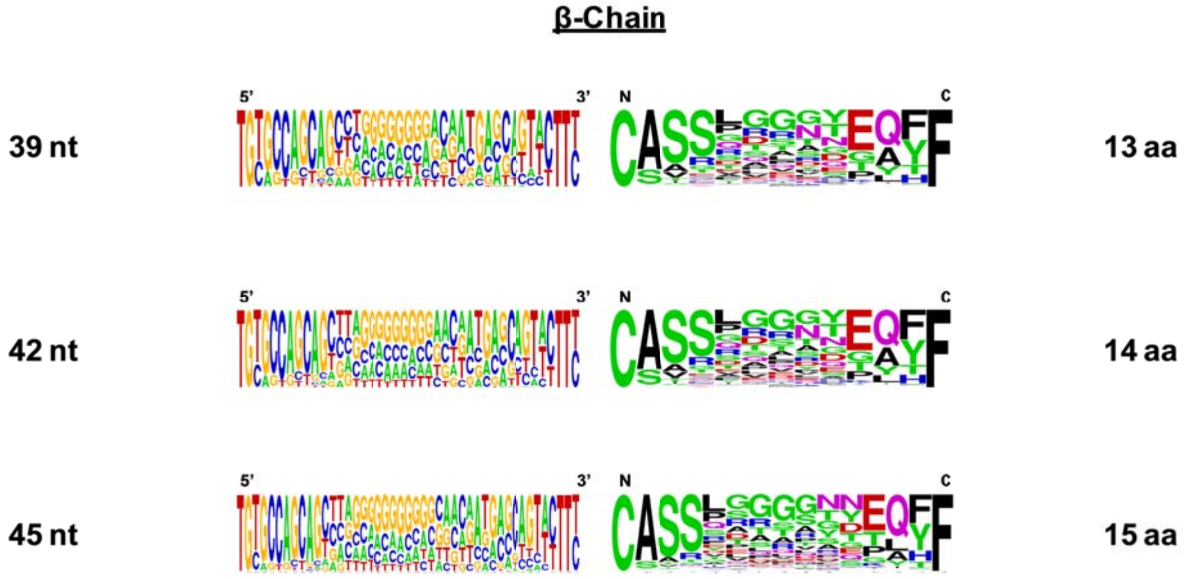

Supplementary Figure 5. CDR3 length and sequence composition. (**a**,**b**) The distribution of the length for the CDR3 clonotypes identified in the 6 HD is represented in the histograms (**a**, α-chain; **b**, β-chain). Every colour indicates a different donor. Results show that there are preferred CDR3 lengths for both chains. (**c, d**) Web Logo graphics[1] for the nt and aa CDR3 sequences of the 6 HD analysed (**c**, α-chain; **d**, β-chain). Overall, CDR3 sequences of the 6 HD with a length ranging between 13 and 15 aa and the corresponding nt sequences have been considered. Results show a high similarity in the nt and aa composition independently of the sequence length for both chains. For each donor, two replicates have been performed and the sequencing results have been combined. CDR3, complementarity determining region 3; HD, healthy donor; nt, nucleotide; aa, amino acid; N, N-terminus; C, C-terminus.

Supplementary Figure 6

a **b**

α- Chain

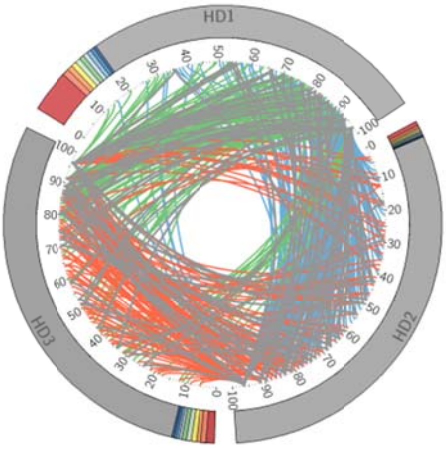

Public Clones HD1-3

HD1-HD2	225
HD1-HD3	173
HD2-HD3	193
HD1-HD2-HD3	35
HD1 vs HD2+HD3	433

% TCR Clones in Public Seq	
HD1	5.2
HD2	5.6
HD3	5.8

Public Clones HD4-6

HD4-HD5	33
HD4-HD6	22
HD5-HD6	41
HD4-HD5-HD6	1

% TCR Clones in Public Seq	
HD4	1
HD5	1.5
HD6	1.4

c **d**

ß-Chain

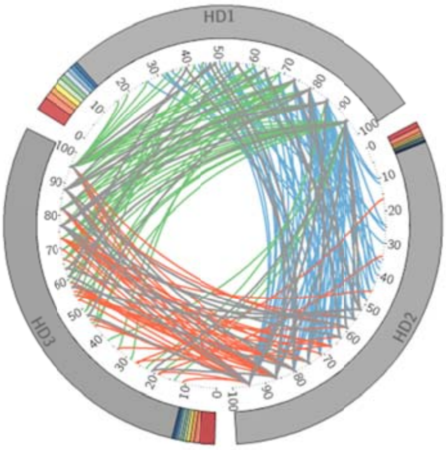

Public Clones HD1-3

HD1-HD2	116
HD1-HD3	84
HD2-HD3	59
HD1-HD2-HD3	13
HD1 vs HD2+HD3	213

% TCR Clones in Public Seq	
HD1	1.4
HD2	1.7
HD3	2.2

Public Clones HD4-6

HD4-HD5	9
HD4-HD6	4
HD5-HD6	2
HD4-HD5-HD6	-

% TCR Clones in Public Seq	
HD4	0.4
HD5	0.2
HD6	0.1

Supplementary Figure 6. Public clones in healthy donors. Circos[2] pictures represent the clonal contributions (retrieval frequency) of CDR3 amino acid (aa) sequences to the TCR repertoire of HD1-3 for the α- (**a**) and β-chain (**c**). The 10 most predominant CDR3 aa clonotypes identified from each donor are represented in the graphic. Each bar represents an individual CDR3 aa clonotype, with red and violet indicating the first and tenth most predominant sequences, respectively. Grey bars indicate the remaining sequences identified in the analysed sample. Each coloured line indicates a public clone and its position on the circular bars reflects its contribution to the TCR repertoire. The number of public clones and the percentage of TCR clones involved in the generation of the public sequences are indicated for HD1-3 in panels **a** (α-chain) and **c** (β-chain) and for HD4-6 in panels **b** (α-chain) and **d** (β-chain). For each donor, two replicates have been performed and the sequencing results have been combined. HD, healthy donor; vs., versus; Seq, sequences.

Supplementary Figure 7

a

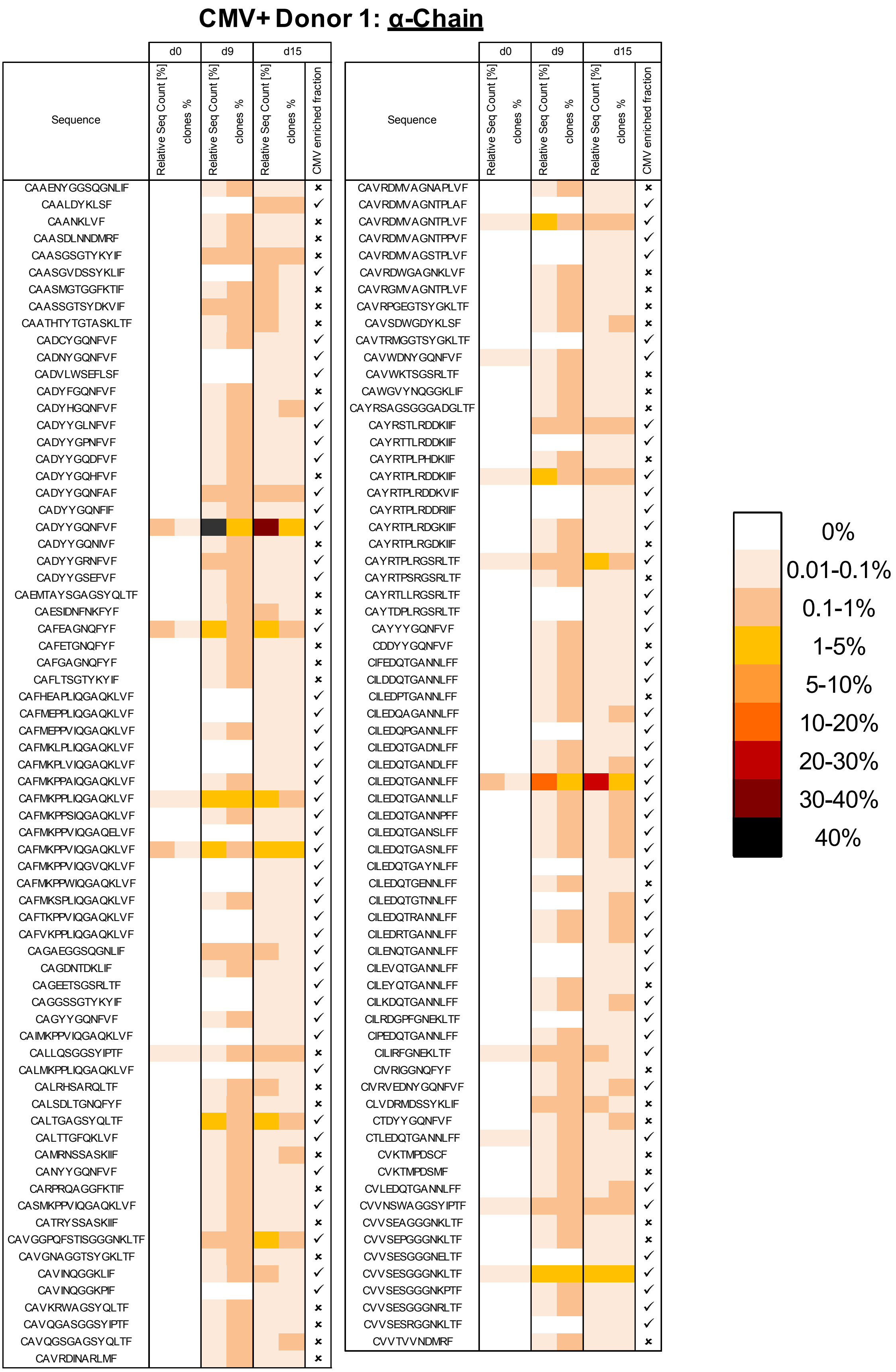

b

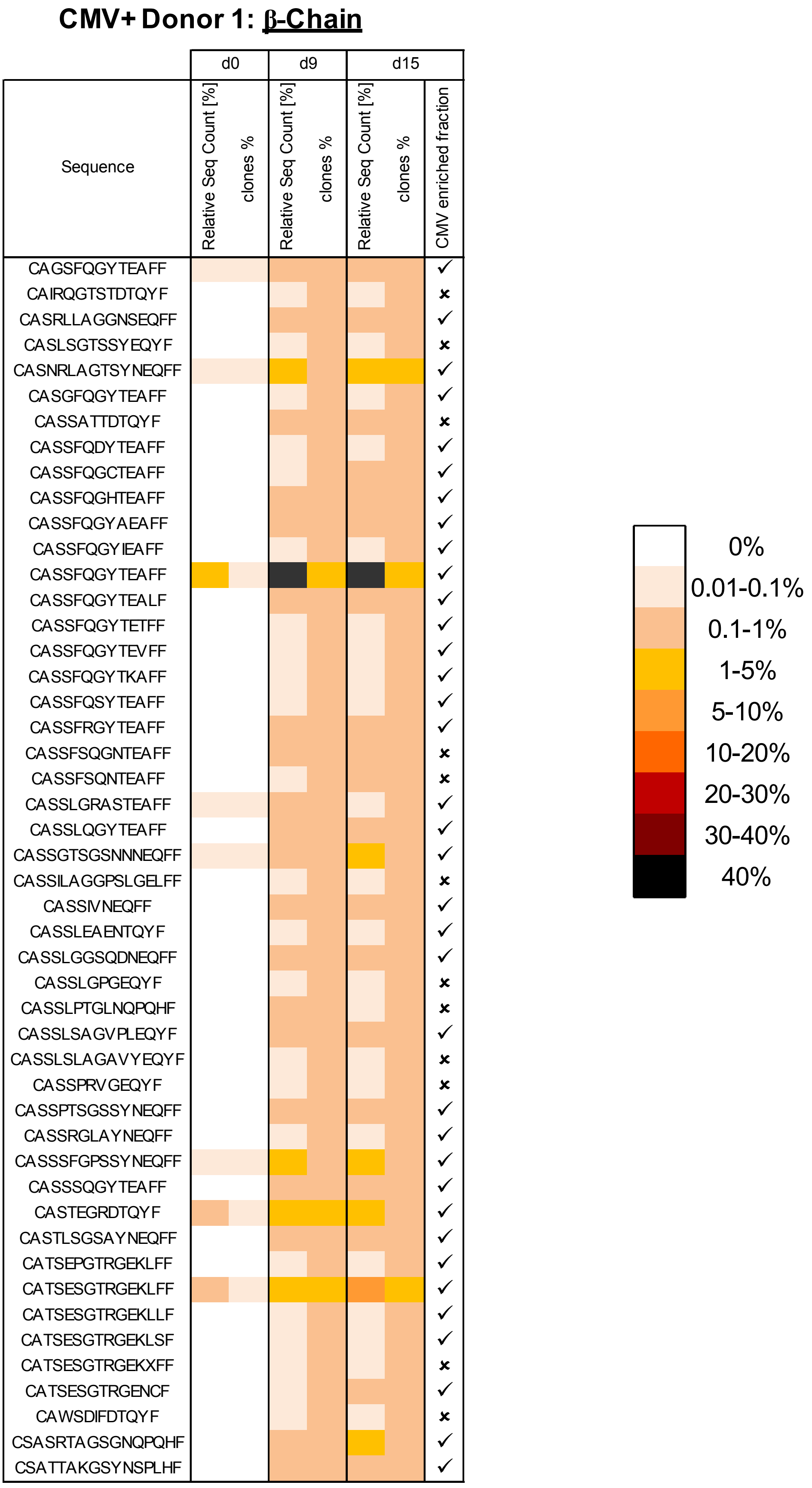
CMV+ Donor 1: β-Chain
d0
d9
d15
Sequence
Relative Seq Count [%]
clones %
CMV enriched fraction
CAGSFQGYTEAFF ✓
CAIRQGTSTDTQYF ×
CASRLLAGGNSEQFF ✓
CASLSGTSSYEQYF ×
CASNRLAGTSYNEQFF ✓
CASGFQGYTEAFF ✓
CASSATTDTQYF ×
CASSFQDYTEAFF ✓
CASSFQGCTEAFF ✓
CASSFQGHTEAFF ✓
CASSFQGYAEAFF ✓
CASSFQGYIEAFF ✓
CASSFQGYTEAFF ✓
CASSFQGYTEALF ✓
CASSFQGYTETFF ✓
CASSFQGYTEVFF ✓
CASSFQGYTKAFF ✓
CASSFQSYTEAFF ✓
CASSFRGYTEAFF ✓
CASSFSQGNTEAFF ×
CASSFSQNTEAFF ×
CASSLGRASTEAFF ✓
CASSLQGYTEAFF ✓
CASSGTSGSNNNEQFF ✓
CASSILAGGPSLGELFF ×
CASSIVNEQFF ✓
CASSLEAENTQYF ✓
CASSLGGSQDNEQFF ✓
CASSLGPGEQYF ×
CASSLPTGLNQPQHF ×
CASSLSAGVPLEQYF ✓
CASSLSLAGAVYEQYF ×
CASSPRVGEQYF ×
CASSPTSGSSYNEQFF ✓
CASSRGLAYNEQFF ✓
CASSSFGPSSYNEQFF ✓
CASSSQGYTEAFF ✓
CASTEGRDTQYF ✓
CASTLSGSAYNEQFF ✓
CATSEPGTRGEKLFF ✓
CATSESGTRGEKLFF ✓
CATSESGTRGEKLLF ✓
CATSESGTRGEKLSF ✓
CATSESGTRGEKXFF ×
CATSESGTRGENCF ✓
CAWSDIFDTQYF ×
CSASRTAGSGNQPQHF ✓
CSATTAKGSYNSPLHF ✓
0%
0.01-0.1%
0.1-1%
1-5%
5-10%
10-20%
20-30%
30-40%
40%

c

CMV+ Donor 2: α-Chain

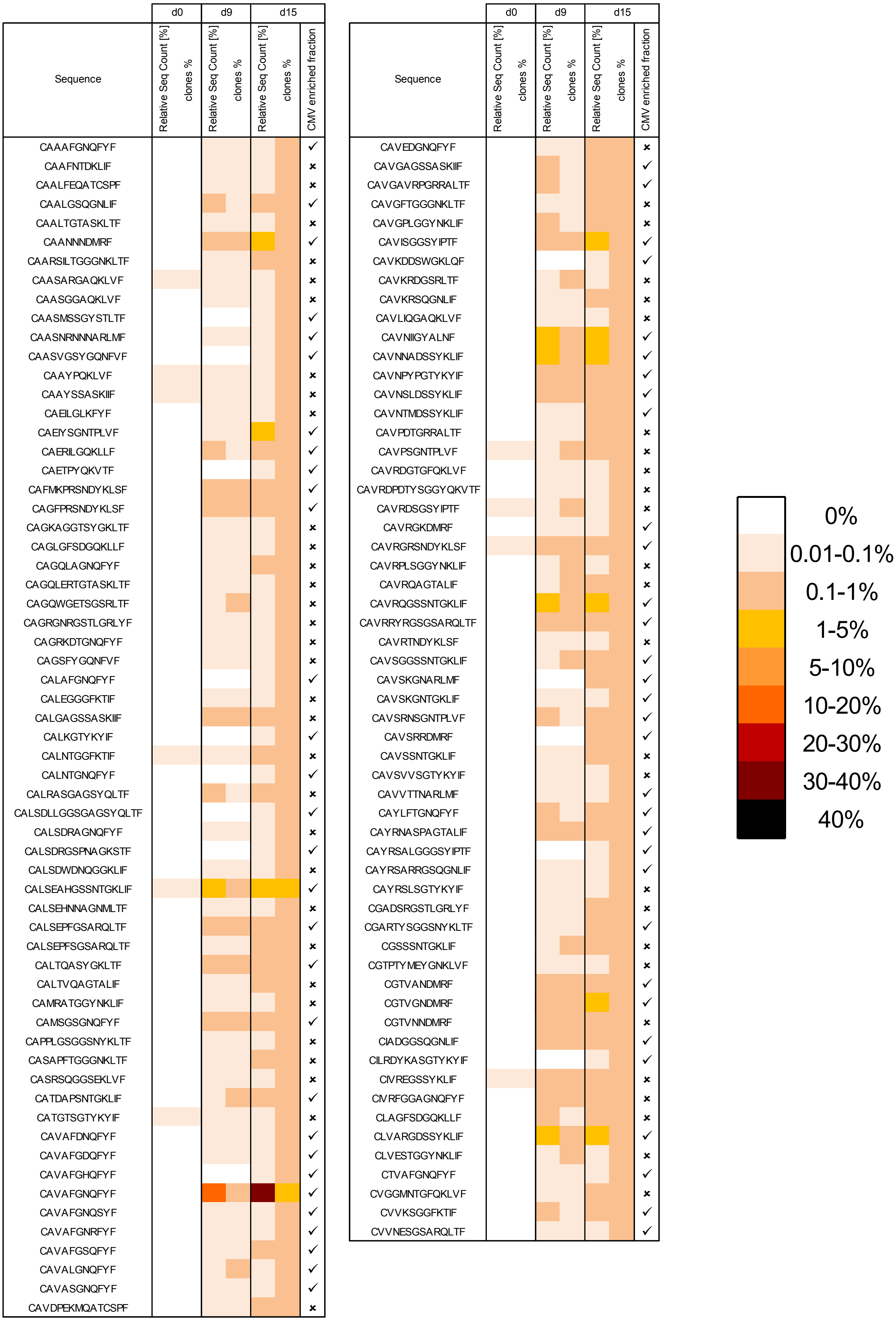

d

CMV+ Donor 2: β-Chain

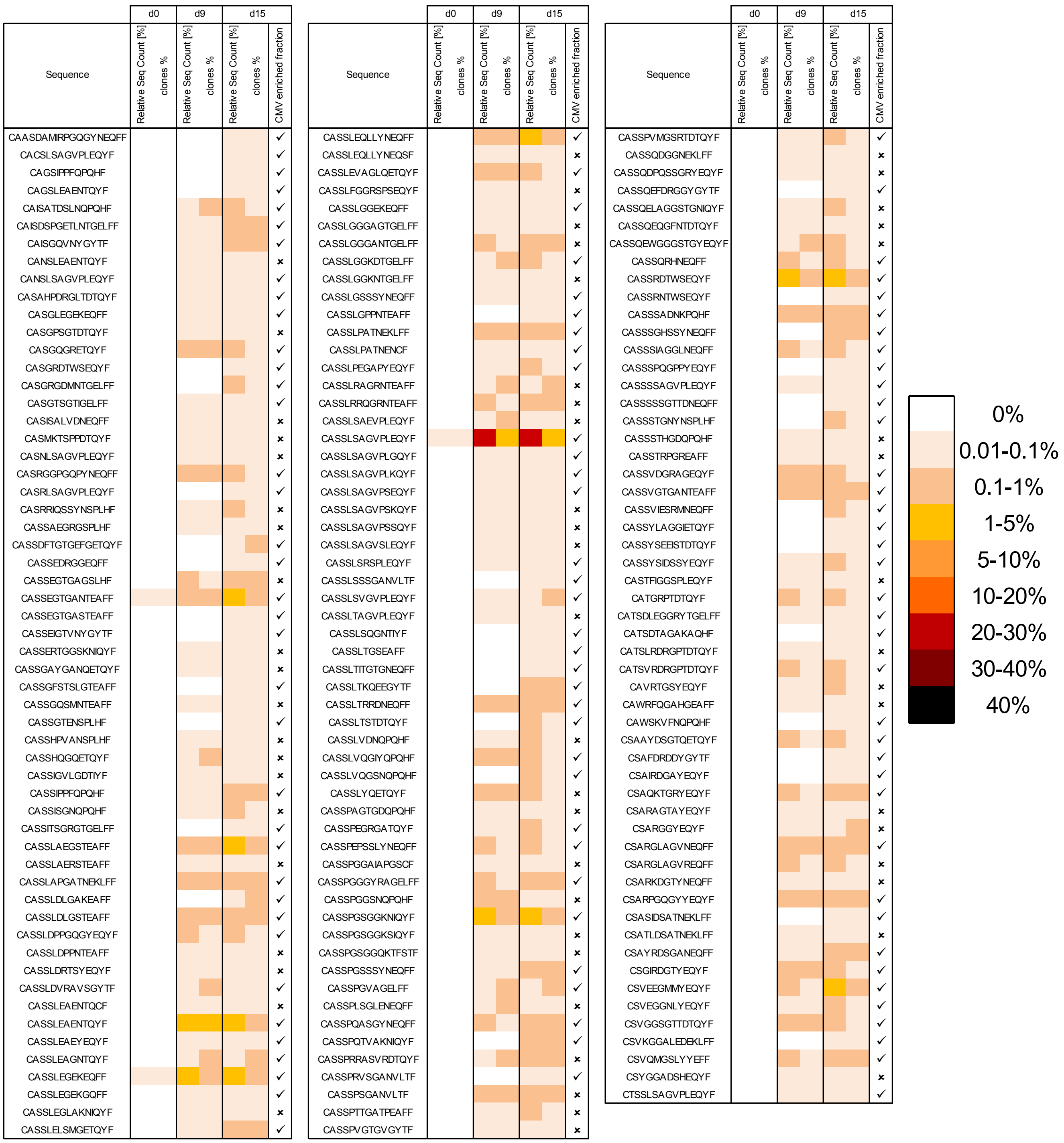

e

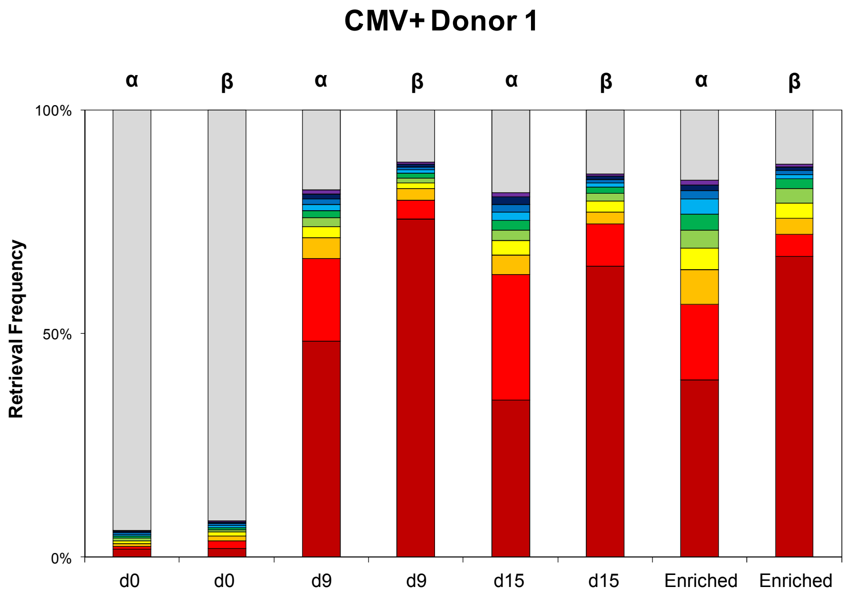

	d0		d9		d15		Enriched	
SI	α	β	α	β	α	β	α	β
	1,194	795	3.7	1.7	4.8	2.3	5.0	2.2

	d0		d9		d15		Enriched	
SA	α	β	α	β	α	β	α	β
	3.5	3.5	1.1	0.7	1.2	0.9	1.1	0.8

f

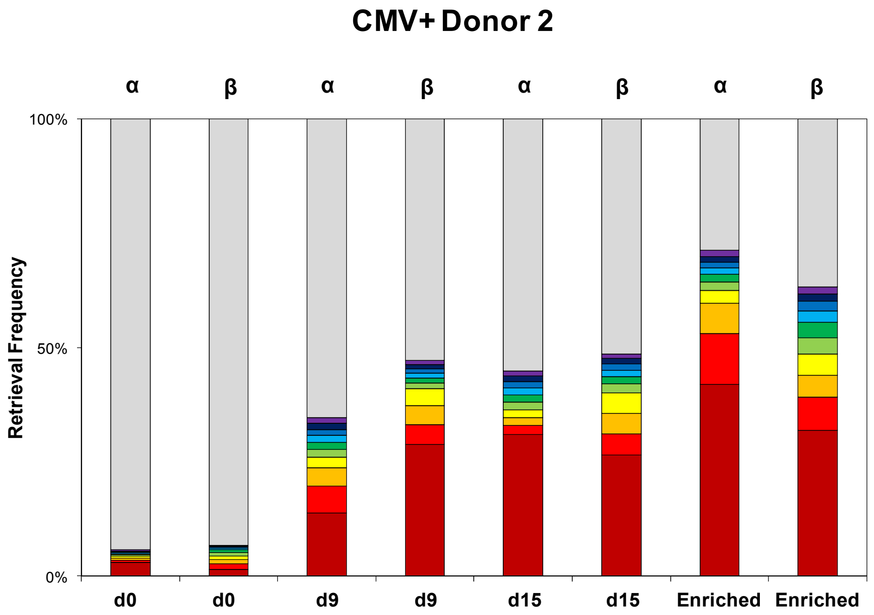

	d0		d9		d15		Enriched	
SI	α	β	α	β	α	β	α	β
	818	1080	36	11.2	10.1	12.7	5.1	8.6

	d0		d9		d15		Enriched	
SA	α	β	α	β	α	β	α	β
	3.5	3.4	2.3	2.0	1.9	2.0	1.3	1.6

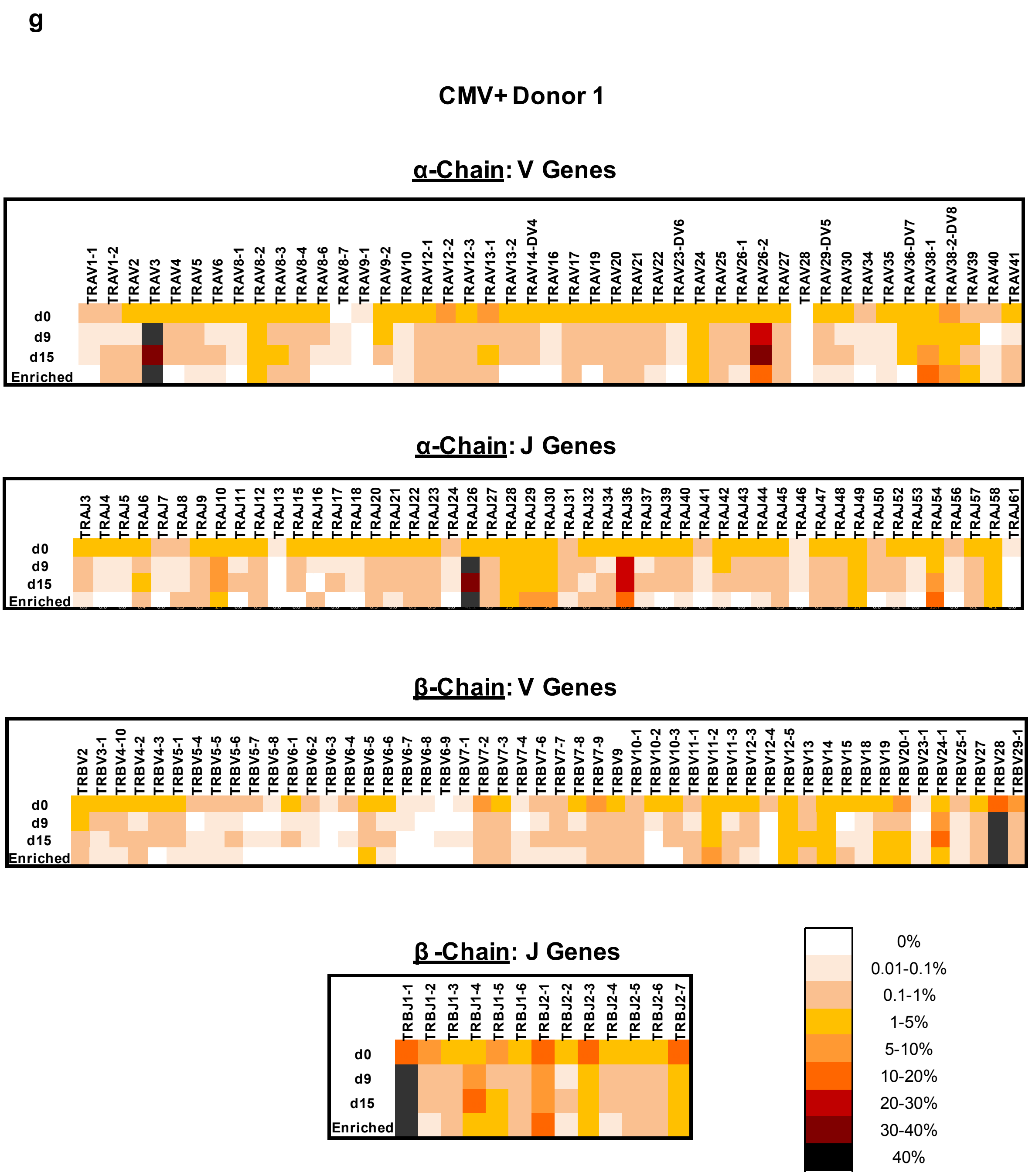
g
CMV+ Donor 1
α-Chain: V Genes
α-Chain: J Genes
β-Chain: V Genes
β -Chain: J Genes
d0
d9
d15
Enriched
0%
0.01-0.1%
0.1-1%
1-5%
5-10%
10-20%
20-30%
30-40%
40%

h

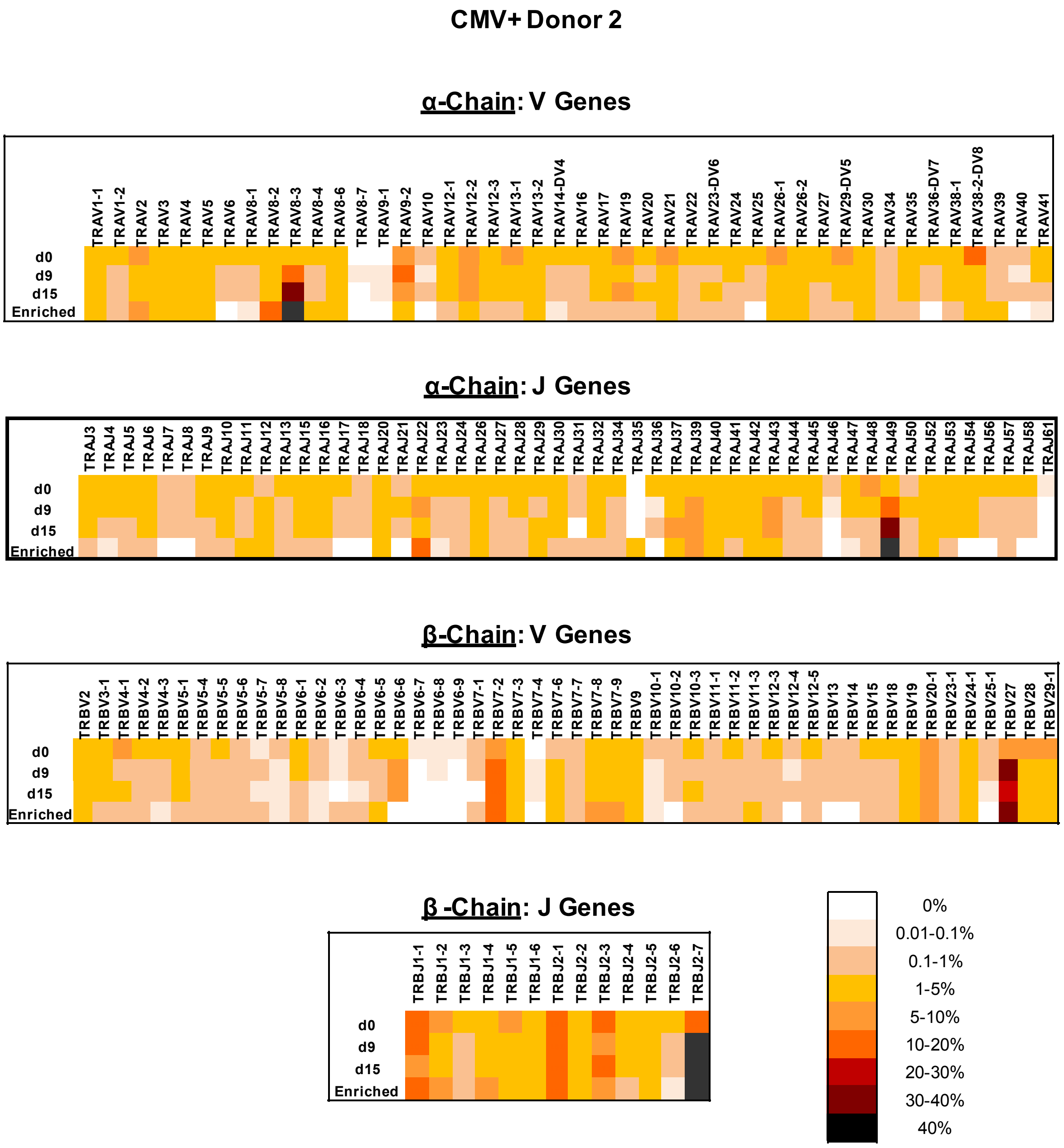
CMV+ Donor 2
α-Chain: V Genes
TRAV1-1 TRAV1-2 TRAV2 TRAV3 TRAV4 TRAV5 TRAV6 TRAV8-1 TRAV8-2 TRAV8-3 TRAV8-4 TRAV8-6 TRAV8-7 TRAV9-1 TRAV9-2 TRAV10 TRAV12-1 TRAV12-2 TRAV12-3 TRAV13-1 TRAV13-2 TRAV14-DV4 TRAV16 TRAV17 TRAV19 TRAV20 TRAV21 TRAV22 TRAV23-DV6 TRAV24 TRAV25 TRAV26-1 TRAV26-2 TRAV27 TRAV29-DV5 TRAV30 TRAV34 TRAV35 TRAV36-DV7 TRAV38-1 TRAV38-2-DV8 TRAV39 TRAV40 TRAV41
d0
d9
d15
Enriched
α-Chain: J Genes
TRAJ3 TRAJ4 TRAJ5 TRAJ6 TRAJ7 TRAJ8 TRAJ9 TRAJ10 TRAJ11 TRAJ12 TRAJ13 TRAJ15 TRAJ16 TRAJ17 TRAJ18 TRAJ20 TRAJ21 TRAJ22 TRAJ23 TRAJ24 TRAJ26 TRAJ27 TRAJ28 TRAJ29 TRAJ30 TRAJ31 TRAJ32 TRAJ34 TRAJ35 TRAJ36 TRAJ37 TRAJ39 TRAJ40 TRAJ41 TRAJ42 TRAJ43 TRAJ44 TRAJ45 TRAJ46 TRAJ47 TRAJ48 TRAJ49 TRAJ50 TRAJ52 TRAJ53 TRAJ54 TRAJ56 TRAJ57 TRAJ58 TRAJ61
d0
d9
d15
Enriched
ß-Chain: V Genes
TRBV2 TRBV3-1 TRBV4-1 TRBV4-2 TRBV4-3 TRBV5-1 TRBV5-4 TRBV5-5 TRBV5-6 TRBV5-7 TRBV5-8 TRBV6-1 TRBV6-2 TRBV6-3 TRBV6-4 TRBV6-5 TRBV6-6 TRBV6-7 TRBV6-8 TRBV6-9 TRBV7-1 TRBV7-2 TRBV7-3 TRBV7-4 TRBV7-6 TRBV7-7 TRBV7-8 TRBV7-9 TRBV9 TRBV10-1 TRBV10-2 TRBV10-3 TRBV11-1 TRBV11-2 TRBV11-3 TRBV12-3 TRBV12-4 TRBV12-5 TRBV13 TRBV14 TRBV15 TRBV18 TRBV19 TRBV20-1 TRBV23-1 TRBV24-1 TRBV25-1 TRBV27 TRBV28 TRBV29-1
d0
d9
d15
Enriched
ß -Chain: J Genes
TRBJ1-1 TRBJ1-2 TRBJ1-3 TRBJ1-4 TRBJ1-5 TRBJ1-6 TRBJ2-1 TRBJ2-2 TRBJ2-3 TRBJ2-4 TRBJ2-5 TRBJ2-6 TRBJ2-7
d0
d9
d15
Enriched
0%
0.01-0.1%
0.1-1%
1-5%
5-10%
10-20%
20-30%
30-40%
40%

Supplementary Figure 7. TCR clonotypes activation in 2 CMV positive donors after stimulation with pp65 and IE-1 peptide pools. Figures **a**, **b** (CMV+ Donor 1) and **c**, **d** (CMV+ Donor 2) represent CMV reactive αβ TCR sequences and their clonal contribution. CDR3 aa clonotypes showing an increase at day 9 and a stable or increasing contribution at day 15 were considered CMV reactive. Reactive TCR present at day 15 could be backtracked on day 0 (up to 16%) and day 9 (up to 100%). Number of clones (in percentage) contributing to the generation of each aa specificity is also reported. Retrieval frequency ranged from 0.01-0.1% (light pink) to >40% (black). CMV reactive T-cells were enriched via IFNγ capture assay. ✓, sequence was identified in the CMV enriched fraction; ×, sequence was not identified in the CMV enriched fraction. T-cell clone numbers related to those CMV sequences increased over time. Figures **e** (CMV+ Donor 1) and **f** (CMV+ Donor 2) represent the TCR sequence contribution for both TCR chains at different time points after stimulation with CMV peptide pools and in the enriched IFNγ-secreting fraction. Each bar represents an individual CDR3 aa clonotype, with red and violet indicating the first and tenth most predominant aa sequence. Grey bars indicate the remaining identified sequences. TCR clonality decreased and specific CDR3 sequences emerged. Figures **g** (CMV+ Donor 1) and **h** (CMV+ Donor 2) show the skewing of *V* and *J* gene usage observed starting from day 9 in both donors and for both TCR chains. Retrieval frequencies of the individual genes are reported in different colours: the darker the colour, the stronger is the usage of a defined gene in the analysed TCR repertoire. For day 0, day 9 and day 15 of each donor, two replicates have been performed and the sequencing results have been combined. d, day; aa, amino acid; *SI*, Simpson diversity index; *SA*, Shannon diversity index; *V*, variable; *J*, joining; CDR3, complementarity determining region 3; *TRAV*, TCR α-chain variable gene; *TRAJ*, TCR α-chain joining gene; *TRBV*, TCR β-chain variable gene; *TRBJ*, TCR β-chain joining gene.

Supplementary Figure 8

a

α -Chain

	TCR Seq	V	J	C	VJ	CDR3 (nt)	CDR3 (aa)
**HD5 PBMC**	265,589	44	48	1	955	11,512	9,826
**CD4 RO-**	40,661	26	26	1	69	938	623
**CD4 RO+**	20,610	51	24	1	47	765	477
**CD8 RO-**	57,983	13	14	1	27	1,289	811
**CD8 RO+**	48,615	8	3	1	5	252	170

b

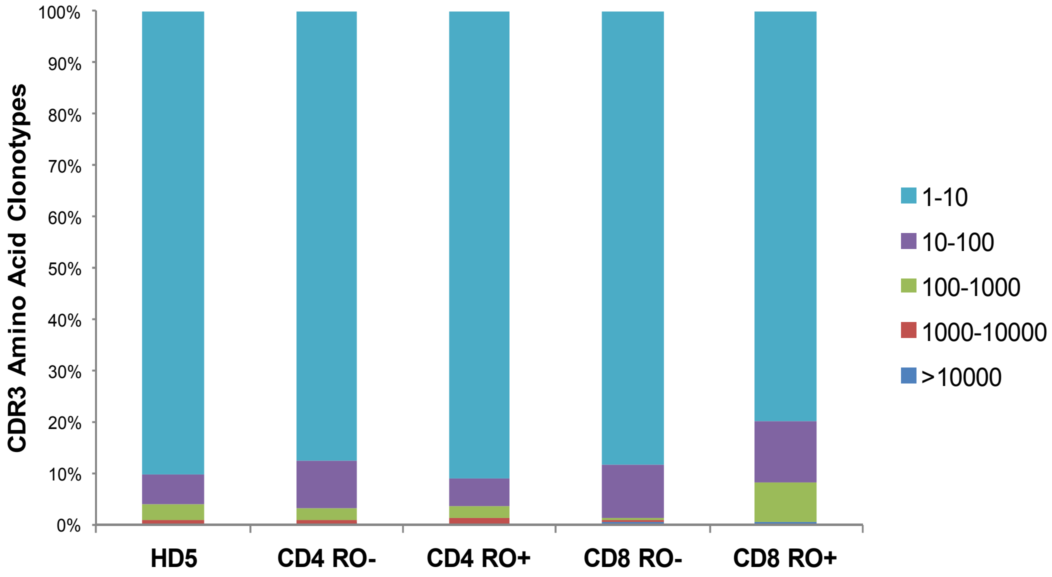

c

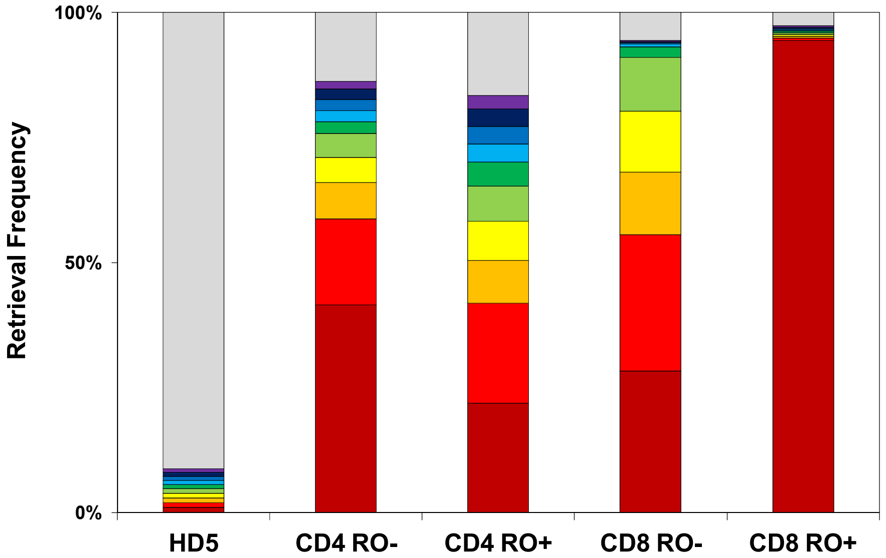

d

β-Chain

	TCR Seq	V	D	J	C	VJ	CDR3 (nt)	CDR3 (aa)
**HD5 PBMC**	205,538	50	2	13	2	470	14,316	9,664
**CD4 RO-**	146,423	34	2	9	2	63	1.825	1,040
**CD4 RO+**	6,676	9	2	5	2	14	177	90
**CD8 RO-**	10,420	25	2	6	2	49	456	234
**CD8 RO+**	8,478	3	2	2	2	5	187	78

e

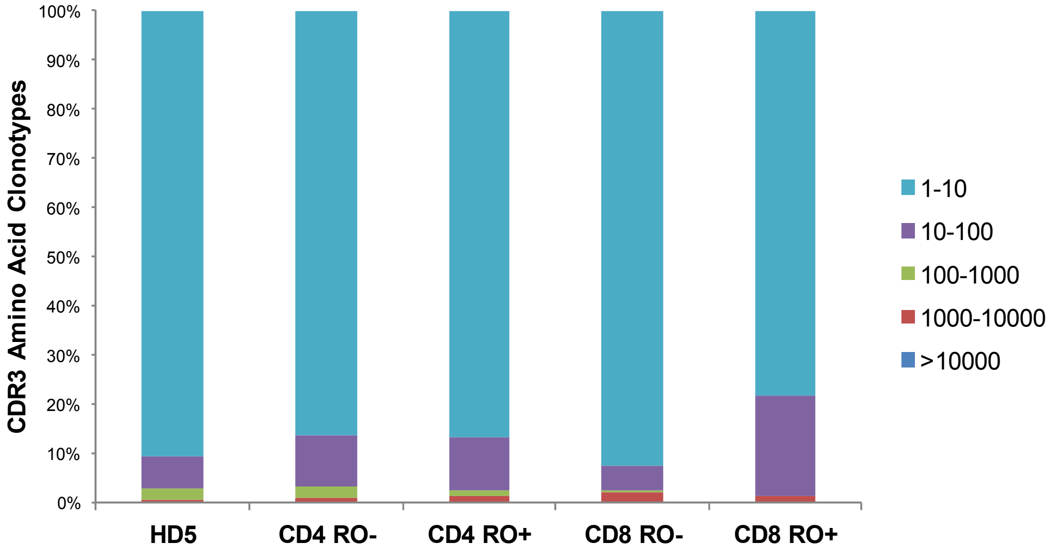

f

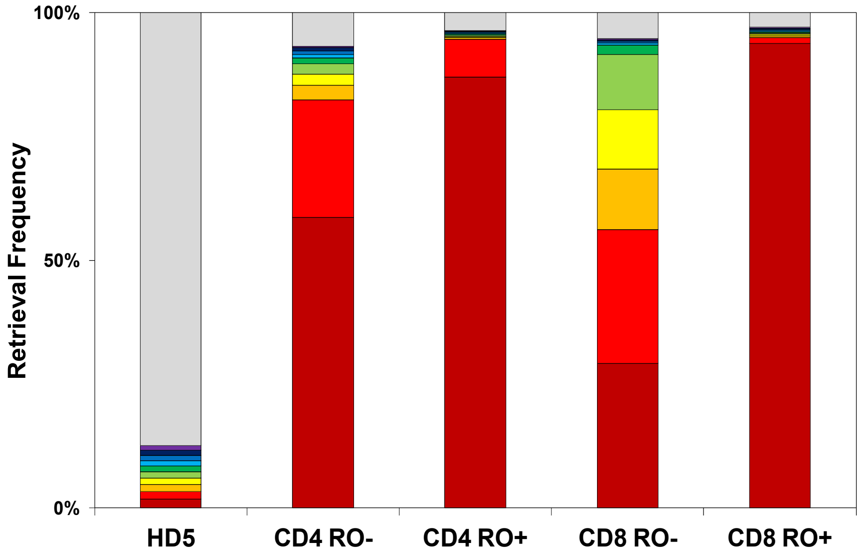

Supplementary Figure 8. TCR repertoire analysis for T-cell subsets. T-cells subsets (CD4RO-, CD4RO+, CD8RO-, CD8RO+) of HD5 were isolated by FACS sorting and analysed via TCR-LA-MC PCR for both TCR chains. Figures **a** (α-chain) and **d** (β-chain) show an overview of the sequencing results. Figures **b** (α-chain) and **e** (β-chain) show the percentage of CDR3 aa clonotypes presenting the number of reads indicated in the different colours. Figures **c** (α-chain) and **f** (β-chain) show the contribution of the 10 most predominant CDR3 aa clones to the TCR repertoire. Each bar represents an individual CDR3 aa clonotype, with red and violet indicating the first and tenth most predominant sequence. Grey bars indicate the contribution of the remaining sequences identified in the analysed sample. Results showed that the CD8 memory compartment is characterized by a major expansion of a few dominant clones compared to the CD4 memory subset. Interestingly, when looking at the CDR3 aa clonotype contributions in the different samples, results show the clear predominance of a single clonotype in the CD8 memory compartment that accounts for ~90% of the retrieved TCR sequences. Overall, our results show that ~80% of CD4 and CD8 memory compartments are made of minimally expanded aa clonotypes complemented by a few highly expanded clonotypes. TCR-LA-MC PCR, TCR ligation anchored-magnetically captured PCR; *V*, variable; *J*, joining; CDR3, complementarity determining region 3; aa, amino acid; nt, nucleotide; TCR Seq, sequences where the CDR3 region was identified.

Supplementary Figure 9

α-Chain

a

Sample	TCR Seq	*V*	*J*	*C*	*V-J*	CDR3 (nt)	CDR3 (aa)
Mouse	41,033	51	38	1	1,403	9,924	8,386

c

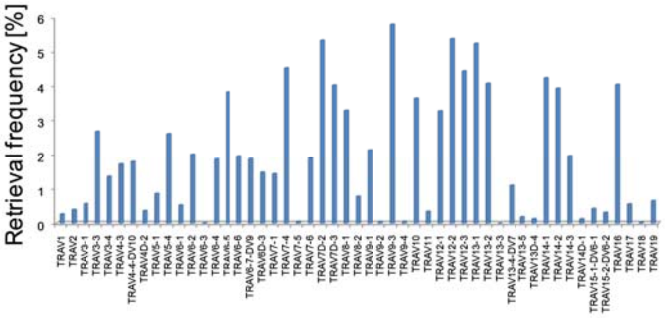

e

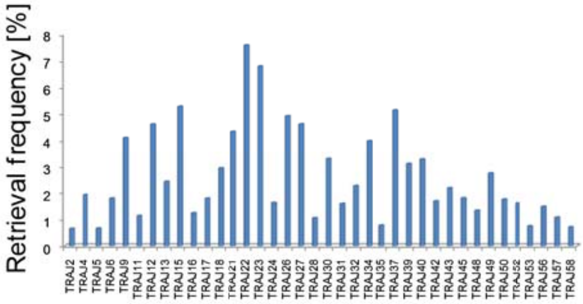

g

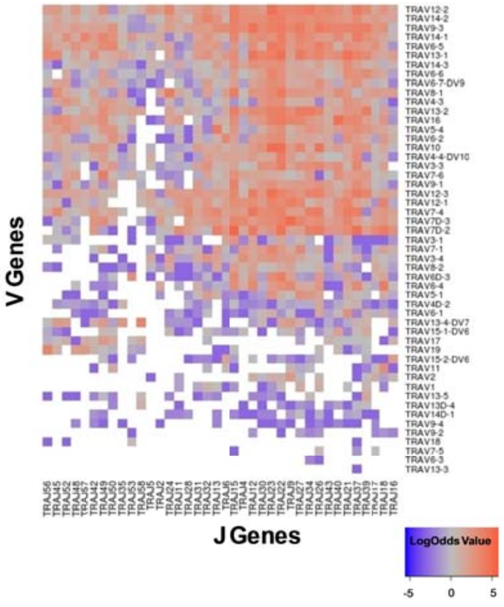

β-Chain

b

Sample	TCR Seq	*V*	*D*	*J*	*C*	*V-J*	CDR3 (nt)	CDR3 (aa)
Mouse	57,933	22	2	12	2	1,586	24,398	22,045

d

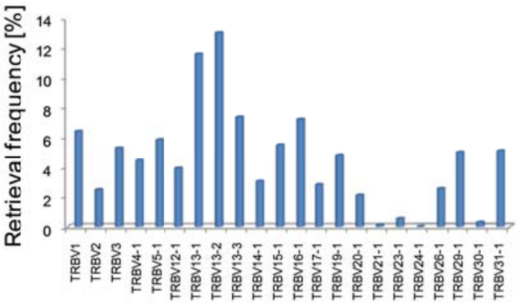

f

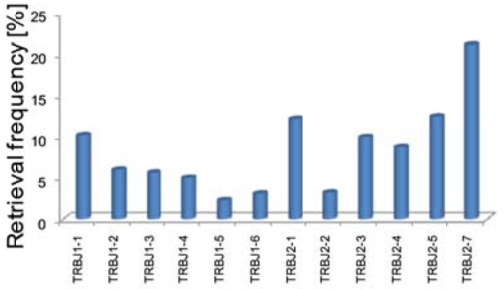

h

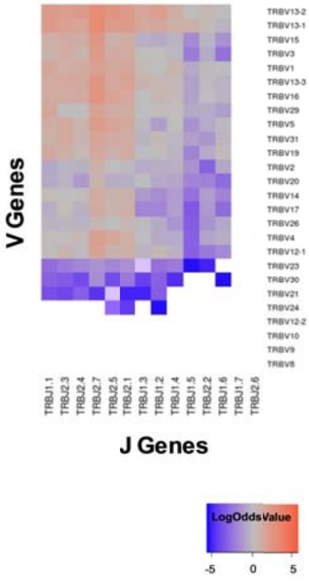

i

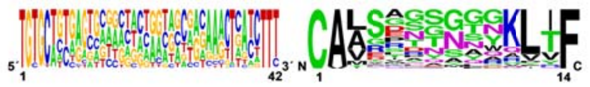

j

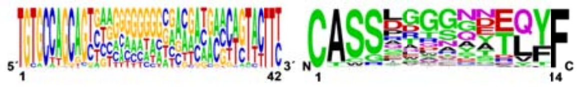

Supplementary Figure 9. TCR diversity in mouse. TCR-LA-MC PCR was performed on RNA isolated from the spleen of a mouse. **a** (α-chain) and **b** (β-chain) show a summary of the results retrieved from the sequencing run. The number of *V* and *J* genes, *VJ* pairings, CDR3 nucleotide and amino acid sequences identified are shown. *V* and *J* gene usage is represented for α- (**c** and **e**) and β-chain (**d** and **f**) as histogram pictures showing a non uniform pattern. *VJ* pairings (**g,** α-chain and **h,** β-chain) are represented as log-odds matrices and show the non randomness in the combinatorial process of the TCR diversity. In blue and in red rarely found and overrepresented pairings are shown, respectively. Each coloured cell represents a defined *VJ* pairing. CDR3 length distribution showed that there was a preferential abundance of 42 nt long sequences. Web logo graphics[1] for the nt and the aa sequences of the CDR3 regions harboring this preferred length are represented for both chains (**i** and **j**). Left side: α-chain results; right side: β-chain results. TCR-LA-MC PCR, TCR ligation anchored-magnetically captured PCR; TCR Seq, sequences where the CDR3 region was identified; *V*, variable; *J*, joining; CDR3, complementarity determining region 3; nt, nucleotide; aa, amino acid; *TRAV*, TCR α-chain variable gene; *TRAJ*, TCR α-chain joining gene; *TRBV*, TCR β-chain variable gene; *TRBJ*, TCR β-chain joining gene.

Supplementary Table 1

a

**Method**	**Sample**	**Input**	**ng**	**TCR Seq**	***V***	***J***	***SA***
MP1	T-cells	DNA	100	28	15	7	1.3
MP1	T-cell clone	DNA	100	188	1	1	0.2
MP2	T-cells	DNA	100	70	11	5	1.0
MP2	PBMC	DNA	100	28	10	5	1.4
MP2	T-cell clone	DNA	100	54	1	1	0.1
MP3	T-cells	DNA	100	399	19	9	2.1
MP3	PBMC	DNA	100	287	16	8	1.8
MP3	T-cell clone	DNA	100	2	1	1	0.0
MP4	PBMC	DNA	100	747	30	11	1.9
MP4	T-cell clone	DNA	100	332	1	1	0.2
MP5	PBMC	DNA	100	71	14	7	1.8
MP5	T-cells	DNA	100	339	22	8	1.3
MP5	PBMC	DNA	100	40	7	4	0.5
MP5	T-cell clone	DNA	100	270	1	1	0.3
MP6	T-cells	DNA	100	475	27	9	1.5
MP6	PBMC	DNA	100	957	16	7	1.0
MP6	T-cell clone	DNA	100	20	1	1	0.2
**TCR-LA-MC PCR**	PBMC	RNA	100	1,086	**42**	**13**	**2.4**
**TCR-LA-MC PCR**	PBMC	RNA	1,000	642	**44**	**13**	**2.6**
RACE PCR	T-cells	RNA	1,000	1,141	12	13	2.7
RACE PCR	T-cells*	RNA	1,000	1,578	5	13	2.3
TCR Express Kit	T-cells	RNA	~2,000	51,219	44	13	2.7
MP2	T-cells	RNA	100	134	17	5	1.6
MP2	PBMC	RNA	100	18	5	5	1.2
MP4	T-cells	RNA	100	21	11	5	1.2
MP5	T-cells	RNA	100	76	14	5	1.5
MP5	PBMC	RNA	100	27	9	4	1.3

b

Protocol	RACE PCR	TCR-LA-MC PCR
cDNA synthesis	Constant gene (*C*)-specific	Constant gene (*C*)-specific (biotinylated) primer
Reverse transcriptase	SmartScribe	Superscript II
Template switching (TSW)	Yes	No
cDNA magnetic capture	No	Yes
Ligation	No	Single-stranded ligation of a known linker cassette (LK)
First exponential PCR	*C* gene-specific primer + TSW primer	*C* gene-specific primer (biotinylated) + LK primer
Magnetic capture	No	Yes
Second exponential PCR	*C* gene-specific primer + TSW primer	*C* gene-specific primer+ LK primer

c

Sample PBMC1; Tested Parameter: 1) Reverse Transcriptase

	TCR-LA-MC PCR 1	TCR-LA-MC PCR 2	TCR-LA-MC PCR 3	TCR-LA-MC PCR 4	TCR-LA-MC PCR 5	TCR-LA-MC PCR 6
Transcriptase used	**SmartScribe**	**SmartScribe**	**SmartScribe**	**SS II**	**SS II**	**SS II**
Raw Seq	488,337	690,466	388,220	411,438	391,660	429,454
TCR Seq	262,637	390,524	235,879	255,165	193,916	174,024
TCR Seq/Raw Seq (%)	53.8	56.6	60.8	62.1	49.5	40.5
		Average: 57%			Average: 51%	
V genes	47	48	47	47	46	45
J genes	13	13	13	13	13	13
CDR3 (nt)	1,758	2,621	1,528	1,511	1,308	993
		Average: 1,970			Average:1,270	
CDR3 (aa)	1,672	2,505	1,468	1,441	1,232	949

d **Sample: PBMC1; Tested Parameter: 2) Magnetic Capture**

	**RACE PCR**	**RACE PCR (MC after cDNA)**	**RACE PCR (MC after 1 expo)**	**RACE PCR (MC after cDNA and 1 expo)**
Raw Seq	451,539	412,644	352,260	245,487
TCR Seq	145,193	182,879	145,600	157,308
TCR Seq/Raw Seq (%)	**32.2**	**44.3**	**41.3**	**64**
V	48	48	48	47
J	13	13	13	13
CDR3 nt	8,834	6,051	5,161	2,676
CDR3 aa	8,727	5,986	5,112	2,636

e **Sample: PBMC2; Tested Parameter: 3) Primers**

	**RACE PCR 1**	**RACE PCR 2**	**RACE PCR 3**	**TCR-LA-MC PCR 1**	**TCR-LA-MC PCR 2 (with RACE primers)**	**TCR-LA-MC PCR 3**
Raw reads	401,505	280,529	424,699	175,131	190,246	215,089
TCR Seq	129,196	105,056	174,033	101,867	153,094	155,442
TCRSeq/RawSeq (%)	32.2	37.4	41.0	58.2	80.5	72.3
		Average: 36.9%			Average: 70.3%	
V	48	47	48	42	44	40
J	13	13	13	13	13	13
CDR3 nt	3,607	3,228	3,149	1,317	1,261	1,006
		Average: 3,338			Average:1,194	
CDR3 aa	3,536	3,185	3,102	1,241	1,221	959

Supplementary Table 1. Comparison of different technologies for TCR repertoire sequencing. (**a**) TCR-LA-MC PCR, RACE PCR[3], various multiplex PCR[4] and a commercial kit were tested on DNA or RNA derived from PBMC, T-cells or a T-cell clone of healthy donor samples. Sequencing results focused on *V* and *J* gene retrieval and on CDR3 population heterogeneity (Shannon Diversity Index, *SA*). The higher is the *SA*, the more diverse is the population. For description of the multiplex variants, see material and methods. (**b**) Overview of the RACE PCR[5] and TCR-LA-MC PCR protocols. We investigated the effect of reverse transcriptase (**c**), magnetic capture (**d**) and primers (**e**) on the efficiency of the TCR sequencing procedure. 150 ng RNA derived from PBMC of healthy donor samples were used as starting material. (**c**) Reverse transcriptase. We performed triplicates of the TCR-LA-MC PCR using either Smartscribe RT or Superscript II RT. Smartscribe RT led to a higher retrieval of TCR specific sequences and of unique CDR3 nt sequences. (**d**) Magnetic capture. Magnetic capture was included in the RACE PCR[5] protocol after cDNA synthesis, first exponential PCR and after both cDNA synthesis and first exponential PCR. Results show that introduction of magnetic capture in the RACE PCR approach leads to an increase in the specificity of the retrieved reads. (**e**) Primers. We have performed linker cassette ligation in the TCR-LA-MC PCR using a sequence that allows the usage of the same primers used for RACE PCR[5]. Triplicate analysis showed that the primers used in the RACE PCR[5]

approach increased the sensitivity of TCR-LA-MC PCR. After sequencing, samples were sorted according to the barcode in the *C* gene specific fusion primer and MiTCR[6] was used for the analysis. TCR Seq, sequences where the CDR3 region was identified; ng, nanogram; *V*, variable; *J*, joining; CDR3, complementarity determining region 3; nt, nucleotide; aa, amino acid. MP, multiplex, TCR-LA-MC PCR, TCR ligation anchored-magnetically captured PCR; RACE PCR, rapid amplification of cDNA ends. T-cells*, RNA derived from a pool of T-cells (Miltenyi Biotec); PBMC, peripheral blood mononuclear cells; SmartScribe, SmartScribe reverse transcriptase; SSII, Superscript II reverse transcriptase; RT, reverse transcriptase.

Supplementary Table 2

a

Spike-in Jurkat in T-cells

Jurkat	T-cells	Jurkat Sequence	Retrieval frequency
10,000	90,000	✓	79%
1,000	99,000	✓	20%
100	99,900	✓	5.6%
10	99,990	✓	0.6%
1	99,999	×	0%

Spike-in Jurkat in PBMC

Jurkat	PBMC	Jurkat Sequence	Retrieval frequency
10,000	90,000	✓	44%
1,000	99,000	✓	9.8%
100	99,900	✓	0.5%
10	99,990	✓	0.02%
1	99,999	✓	0.05%

b

***V* Gene**	***J* gene**	**Junction Sequence**	**Junction Amino Acid Sequence**
TRBV25-1	*TRBJ1-1*	TGTGCCAGCAGGGGGTTCACTGAAGCTTTCTTT	CASRGFTEAFF
TRBV12-5	*TRBJ1-1*	TGTGCCAGCAGTCCCACGGGTCGGCAAACTGAAGCTTTCTTT	CASSPTGRQTEAFF
TRBV6-6	*TRBJ2-5*	TGTGCCAGCAGTTACCCGGCGGGGCGCACCCAGTACTTC	CASSYPAGRTQYF

c

	**α-Chain**					
	**MAIT Cells** ***TRAV1-2/TRAJ33*** **- CAVRDSNYQLIW**			**iNKT Cells** ***TRAV10/TRAJ18*** **- CVVSDRGSTLGRLYF**		
	VJ	CDR3	Retrieval Frequency	*VJ*	CDR3	Retrieval Frequency
HD1	✓	CAVRDSNYQLIW	0.005%	✓	CVVSDRGSTLGRLYF	0.001%
HD2	✓	CAV**X**DSNYQLIW	0.1%	✓	CVVSDRGSTLGRLYF	0.001%
HD3	✓	CAV**X**DSNYQLIW	0.08%	-	-	-
HD4	✓	CAVRD**G**NYQLIW	0.01%	✓	CVV**XX**DRGSTLGRLYF	0.006%
HD5	✓	CAV**X**DSNYQLIW	0.6%	✓	CVVS**X**RGSTLGRLYF*	-
HD6	✓	CAVRDSNYQLIW	0.0003%	-	-	-

Supplementary Table 2. TCR-LA-MC PCR performance. (**a**) Different numbers of Jurkat E6.1 cells were spiked-in a polyclonal background (T-cells, left panel or PBMC, right panel). β-chain sequencing identified the Jurkat E6.1 sequence down to single copy. Tables indicate experimental conditions, Jurkat E6.1 TCR sequence identification and its retrieval frequency. Two replicates were performed for each sample and sequencing results were combined. (**b**) TCR-LA-MC PCR for the β-chain was performed on single sorted cells, showing efficiency of 20% in detecting TCR from single cells. (**c**) TCR signatures of mucosal associated invariant T (MAIT) cells and invariant natural killer T (iNKT) cells, 2 T-cell populations harboring an invariant TCR, were identified in HD samples. TCR α-chain is always originated by the rearrangement of *TRAV1-2* and *TRAJ33* for MAIT cells and *TRAV10* and *TRAJ18* for iNKT cells with a consensus CDR3 amino acid sequence (aa) of the α-chain[7, 8]. We identified the known MAIT sequence (CAVRDSNYQLIW) in the PBMC of the 6 HD included in our analysis. In HD1 and 6, the MAIT CDR3 aa sequence was found with 100% identity, in HD2, 3, 4, 5, with one mismatch (labeled in red) . For iNKT cells, the consensus CDR3 amino acid (CVVSDRGSTLGRLYF) of the α-chain was identified in 3 out of 6 HD. In one case, we identified a sequence with 2 mismatches (labeled in red). Retrieval frequencies of the CDR3 α-chain for both MAIT and iNKT cells are reported. *For HD5 we performed MACS separation of the iNKT cells followed by FACS sorting and TCR sequencing. We retrieved the canonical TCR α-chain sequence, we identified the *V* β-chain gene that is known in literature to be the preferred used one as well as a dominant (over 90%) CDR3 β-chain sequence (CASSGAGYNQPQHF). The occurrence of a predominant β-chain sequence has never been shown in previous reports. ✓, Jurkat TCR sequence detected; ×, Jurkat TCR sequence not detected; TCR-LA-MC PCR, TCR ligation anchored-magnetically captured PCR; PBMC, peripheral blood mononuclear cells; HD, healthy donor; *V*, variable; *J*, joining; *TRAV*, TCR α-chain variable gene; *TRAJ*, TCR α-chain joining gene.

Supplementary Table 3

α-Chain

Sample	TCR Seq	*V*	*J*	*C*	*V-J*	CDR3 (nt)	CDR3 (aa)
HD1	118,855	44	50	1	1,586	13,312	11,263
HD2	78,286	44	50	1	1,551	11,422	9,911
HD3	78,895	43	49	1	1,512	10,575	9,161
HD4	183,945	44	48	1	955	11,089	8,020
HD5	265,589	44	50	1	1,053	13,697	9,826
HD6	223,758	42	49	1	940	11,354	8,128

β-Chain

Sample	TCR Seq	*V*	*D*	*J*	*C*	*V-J*	CDR3 (nt)	CDR3 (aa)
HD1	89,368	51	2	13	2	609	20,603	19,161
HD2	59,207	51	2	13	2	585	13,859	12,829
HD3	49,556	51	2	13	2	581	10,300	9,447
HD4	162,179	49	2	13	2	421	9,336	6,791
HD5	205,538	50	2	13	2	470	13,099	9,664
HD6	238,311	51	2	13	2	450	13,053	9,537

Supplementary Table 3. Overview of the TCR diversity in 6 healthy donors. TCR sequencing results for HD1-6. The number of TCR sequences (sequences where the CDR3 region was identified), *V, D, J, C* genes, *VJ* pairings, CDR3 nucleotide (nt) and amino acid (aa) sequences are reported. Two replicates were performed for each sample and sequencing results were combined. HD, healthy donor; TCR Seq, sequences where the CDR3 region was identified; *V*, variable; *D*, diversity; *J*, joining; *C*, constant; CDR3, complementarity determining region 3.

Supplementary Table 4

<u>α- Chain</u>

V genes	HD1	HD2	HD3	HD4	HD5	HD6
TRAV1-1						
TRAV1-2						
TRAV2						
TRAV3						
TRAV4						
TRAV5						
TRAV6						
TRAV7						
TRAV8-1						
TRAV8-2						
TRAV8-3						
TRAV8-4						
TRAV8-5-pseudogene						
TRAV8-6						
TRAV8-7						
TRAV9-1						
TRAV9-2						
TRAV10						
TRAV11-pseudogene						
TRAV12-1						
TRAV12-2						
TRAV12-3						
TRAV13-1						
TRAV13-2						
TRAV14-DV4						
TRAV15-pseudogene						
TRAV16						
TRAV17						
TRAV18						
TRAV19						
TRAV20						
TRAV21						
TRAV22						
TRAV23-DV6						
TRAV24						
TRAV25						
TRAV26-1						
TRAV26-2						
TRAV27						
TRAV28-pseudogene						
TRAV29-DV5						
TRAV30						
TRAV31-pseudogene						
TRAV32-pseudogene						
TRAV33-pseudogene						
TRAV34						
TRAV35						
TRAV36-DV7						
TRAV37-pseudogene						
TRAV38-1						
TRAV38-2-DV8						
TRAV39						
TRAV40						
TRAV41						

J genes	HD1	HD2	HD3	HD4	HD5	HD6
TRAJ1-orf						
TRAJ2-orf						
TRAJ3						
TRAJ4						
TRAJ5						
TRAJ6						
TRAJ7						
TRAJ8						
TRAJ9						
TRAJ10						
TRAJ11						
TRAJ12						
TRAJ13						
TRAJ14						
TRAJ15						
TRAJ16						
TRAJ17						
TRAJ18						
TRAJ19-orf						
TRAJ20						
TRAJ21						
TRAJ22						
TRAJ23						
TRAJ24						
TRAJ25-orf						
TRAJ26						
TRAJ27						
TRAJ28						
TRAJ29						
TRAJ30						
TRAJ31						
TRAJ32						
TRAJ33						
TRAJ34						
TRAJ35-orf						
TRAJ36						
TRAJ37						
TRAJ38						
TRAJ39						
TRAJ40						
TRAJ41						
TRAJ42						
TRAJ43						
TRAJ44						
TRAJ45						
TRAJ46						
TRAJ47						
TRAJ48						
TRAJ49						
TRAJ50						
TRAJ51-pseudogene						
TRAJ52						
TRAJ53						
TRAJ54						
TRAJ55-pseudogene						
TRAJ56						
TRAJ57						
TRAJ58-orf						
TRAJ59-orf						
TRAJ60-pseudogene						
TRAJ61						

β-Chain

V genes	HD1	HD2	HD3	HD4	HD5	HD6
TRBV1-pseudogene						
TRBV2						
TRBV3-1						
TRBV3-2-pseudogene						
TRBV4-1						
TRBV4-2						
TRBV4-3						
TRBV5-1						
TRBV5-2-pseudogene						
TRBV5-3-orf						
TRBV5-4						
TRBV5-5						
TRBV5-6						
TRBV5-7-orf						
TRBV5-8						
TRBV6-1						
TRBV6-2						
TRBV6-3						
TRBV6-4						
TRBV6-5						
TRBV6-6						
TRBV6-7-orf						
TRBV6-8						
TRBV6-9						
TRBV7-1-orf						
TRBV7-2						
TRBV7-3						
TRBV7-4						
TRBV7-5-pseudogene						
TRBV7-6						
TRBV7-7						
TRBV7-8						
TRBV7-9						
TRBV8-1-pseudogene						
TRBV8-2-pseudogene						
TRBV9						
TRBV10-1						
TRBV10-2						
TRBV10-3						
TRBV11-1						
TRBV11-2						
TRBV11-3						
TRBV12-1-pseudogene						
TRBV12-2-pseudogene						
TRBV12-3						
TRBV12-4						
TRBV12-5						
TRBV13						
TRBV14						
TRBV15						
TRBV16						
TRBV17-orf						
TRBV18						
TRBV19						
TRBV20-1						
TRBV21-1-pseudogene						
TRBV22-1-pseudogene						
TRBV23-1-orf						
TRBV24-1						
TRBV25-1						
TRBV26-pseudogene						
TRBV27						
TRBV28						
TRBV29-1						
TRBV30						
TRBVA-pseudogene						

J genes	HD1	HD2	HD3	HD4	HD5	HD6
TRBJ1-1						
TRBJ1-2						
TRBJ1-3						
TRBJ1-4						
TRBJ1-5						
TRBJ1-6						
TRBJ2-1						
TRBJ2-2						
TRBJ2-2P-orf						
TRBJ2-3						
TRBJ2-4						
TRBJ2-5						
TRBJ2-6						
TRBJ2-7						

Supplementary Table 4. *V* and *J* gene usage for alpha- and beta-chains in healthy donors. In the table are indicated the *V* and *J* genes constituting the α- and β-chain locus. Functional genes, open reading frame (ORF) regions and pseudogenes are included. Grey shading denotes genes identified in healthy donors by TCR-LA-MC PCR sequencing. Two replicates were performed for each donor and sequencing results were combined. *TRAV*, TCR α-chain variable gene; *TRAJ*, TCR α-chain joining gene; *TRBV*, TCR β-chain variable gene; *TRBJ*, TCR β-chain joining gene.

Supplementary Table 5

Convergent Recombination	HD1	HD2	HD3	HD4	HD5	HD6
TCR aa sequences originated by convergent recombination	15.8% α<br>12.3% β	15.4% α<br>11.6% β	13.8% α<br>13.6% β	15.2% α<br>17.4% β	15.1% α<br>16.7% β	15.5% α<br>17.1% β
# unique aa seqs originated by different *VJ* pairings	4% α<br>6.5% β	4.2% α<br>5% β	3.5% α<br>6.8% β	3.8% α<br>6.1% β	4% α<br>5.5% β	4% α<br>5.9% β
# unique aa seqs originated by different nt seqs	11.4% α<br>5.8% β	11.2% α<br>6.8% β	10.3% α<br>6.8% β	11.4% α<br>11.3% β	11.1% α<br>11.2% β	11.5% α<br>11.2% β
# unique aa seqs originated by both convergence steps (different *VJ* pairings and different nt seqs)	1.9% α<br>1% β	1.7% α<br>0.8% β	1.7% α<br>1.2% β	2.8% α<br>3.4% β	2.4% α<br>3.3% β	2.7% α<br>4.1% β

Supplementary Table 5. Overview of the convergent recombination events. A detailed analysis of the convergent recombination process has been performed for HD1-6. Results indicate that up to 11% of the aa sequences in both chains were produced through transcriptional convergence, i.e. producing the identical aa combination from different nt sequences, and that an additional 4% of CDR3 α-chain and 6% of CDR3 β-chain aa sequences, respectively, were recombinatorially convergent. In these cases the same CDR3 aa sequence was found to be generated by different *V-J* pairings. On average 2% of the TCR α- and β-chain sequences were generated by both processes of convergent recombination. Two replicates were performed for each sample and sequencing results were combined. HD, healthy donor; aa, amino acid; nt, nucleotide; *V*, variable; *J*, joining.

Supplementary Table 6

Sample	Gender	Age	CMV	*HLA-A*	*HLA-B*	*HLA-C*	*HLA-DRB1*	*HLA-DQB1*	*HLA-DPB1*
HD1	male	65	✓	02:01	07:02	03:03	13.01	06.02	03.01
				03:01	15:01	07:02	15.01	06.03	04.01
HD2	female	26	×	01:01	08:01	03:03	03.01	02.01	03.01
				24:02	15:01	07:01	16.01	05.02	17.01
HD3	male	43	✓	02:01	13:02	02:02	04.01	03.01	04.01
				30:01	27:05	06:02	11.01	03.02	empty
HD4	female	48	×	01:01	08:01	07:01	03.01	02.01	04.01
				empty	empty	empty	04.07	03.01	empty
HD5	female	29	×	01:01	08:01	07:01	12.01	03.01	04.02
				03:01	52:01	12:02	15.02	06.01	empty
HD6	female	29	-	11:01	18:01	02:02	03.01	02.01	06.01
				24:02	27:02	07:02	11.01	03.01	104:01

Supplementary Table 6. HLA typing of the 6 healthy donors. Both *HLA* classes (I: *HLA-A*, *HLA-B*, *HLA-C* and II:*HLA-DRB1*, *HLA-DQB1*, *HLA-DPB1*) were screened. The presence of the same colour in more than one donor indicate that they harbor the same *HLA* allele. Different alleles are labeled in different colours. CMV, cytomegalovirus; HLA, human leukocyte antigen; ✓, CMV seropositive; ×, CMV negative.

Supplementary Table 7

HD

α-Chain							
**Sequence**	**Infection**	**HD1**	**HD2**	**HD3**	**HD4**	**HD5**	**HD6**
CARNTGNQFYF	CMV	0.0076					
CAVYYGQNFVF	CMV				0.0005		
CAMNTGNQFYF	CMV					0.0004	
CAPYTGTASKLTF	CMV					0.0004	
CAGGGSQGNLIF	Influenza						0.0027
CAALGGSQGNLIF	Influenza		0.0026				

β-Chain							
**Sequence**	**Infection**	**HD1**	**HD2**	**HD3**	**HD4**	**HD5**	**HD6**
CASSPPAGSYNEQFF	CMV	0.0011					
CASSLEGYTEAFF	CMV		0.0017				0.0004
CASSANYGYTF	CMV				0.0031		
CASSTNTEAFF	Influenza	0.0022					
CASSLGQAYEQYF	EBV	0.0134			0.3860	0.0482	

Supplementary Table 7. Identification of public clones listed in literature. A list of public sequences published in previous reports[9-14] and identified to be specific for the CMV, EBV and Influenza has been screened in order to identify their presence in our HD samples. Results showed the presence of known public clone sequences in our dataset for both TCR chains. Relative retrieval frequencies of the individual CDR3 amino acid clonotypes identified are indicated in %. Two replicates were performed for each sample and sequencing results were combined. CMV, Cytomegalovirus; EBV, Epstein-Barr virus; HD, healthy donor.

Supplementary Table 8

FACS Results		IFNγ secreting cells [%] CMV+ Donor 1			CMV+ Donor 2		
		d0	d9	d15	d0	d9	d15
Fraction	CD4	0	0.8	0.4	0.1	1.8	3.1
	CD8	1.8	29.7	35.4	0.2	29	22.5

Supplementary Table 8. FACS analysis for IFNγ secretion. Monitoring of the IFNγ secretion was performed for both donors. Results are reported in percentage (%). An increase of IFNγ secretion was detected after stimulation with CMV peptides, expecially in the CD8 fraction of CMV+ Donor 1. CMV, Cytomegalovirus; IFN, interferon; d, day.

Supplementary Table 9

CMV Donors

Sample	CMV	*HLA-A*	*HLA-B*	*HLA-C*	*HLA-DRB1*	*HLA-DQB1*	*HLA-DPB1*
**CMV+ Donor 1**	✓	02:01	27:05	03:03	01:01	03:02	04:01
		23:01	49:01	07:01	04:04	05:01	04:02
**CMV+ Donor 2**	✓	02:01	14:01	04:01	03:01	02:01	01:01
		03:01	35:01	08:02	15:01	06:02	03:01

**α-Chain**									
**Sequence**	**Infection**	**CMV Donor 1**				**CMV Donor 2**			
		**day 0**	**day 9**	**day 15**	**Enriched**	**day 0**	**day 9**	**day 15**	**Enriched**
CAGPMKTSYDKVIF	CMV					✓	✓	✓	✓
CAKNTGNQFYF	CMV						✓		
CARNTGNQFYF	CMV		✓	✓					
CAVAFGNQFYF	CMV						✓	✓	✓
CAVYYGQNFVF	CMV		✓	✓					
CILDNNNDMRF	CMV						✓	✓	✓

**β-Chain**									
**Sequence**	**Infection**	**CMV Donor 1**				**CMV Donor 2**			
		**day 0**	**day 9**	**day 15**	**Enriched**	**day 0**	**day 9**	**day 15**	**Enriched**
CASSLEGYTEAFF	CMV			✓					
CASSSANYGYTF	CMV						✓	✓	✓
CASSLAPGATNEKLFF	CMV						✓	✓	✓
CASSFQGYTEAFF	CMV	✓	✓	✓	✓	✓	✓		

Supplementary Table 9. Identification of TCR public clones specific for the *HLA-02:01* allele. HLA typing results for both CMV seropositive donors showed that both are *HLA-02:01* positive. Thus, we have screened our sequencing datasets in order to identify public sequences known to be specific for the CMV, EBV and Influenza[9-14] and restricted to the *HLA-02:01* allele. Results showed the presence of known public clone sequences in our datasets for both TCR chains. Two replicates were performed for each sample and sequencing results were combined. ✓ Public clone sequence identified.

Supplementary Table 10

Patient	Gender, Age	TNM	Leukocyte/nl	T Lymphocytes	CD4/µl	Sezary Cell Count/µl	CD4/CD8	Skin Involvement	% Skin Involved
1	m, 68	T4NxM0B2	7.94	92.9%	2.409	2.195	61.3	severe	100
2	m, 61	T4NxM0B2	5.29	75.5%	416	324	3.3	severe	100
3	f, 63	T4NxM0B2	4.84	66.1%	207	15	1.8	mild	<20
4	f, 66	T4NxM0B2	11.82	94.9%	4,840	4.882	35.6	severe	100
5	f, 67	T4NxM0B2	9.23	85.7%	806	75	8.8	severe	100
6	m, 52	T4NxM0B2	4.71	48.8%	408	730	2.1	mild	<20
7	m, 67	T4NxM0B2	5.31	79.6%	547	171	1.7	mild	50
8	m, 69	T4NxM0B2	7.05	90.9%	2.512	380	7.9	mild	50
9	m, 52	T4NxM0B2	5.72	79.4%	945	752	3.6	severe	>80
10	m, 65	T4NxM0B2	6.62	91.6%	1.044	817	5.1	severe	>80

Normal Value			4,2 - 10,2	60 - 83%	528 - 1495		1 - 2,8		

Supplementary Table 10. Clinical data for 10 Sézary patients included in our analysis. Values from healthy donor status (normal value) are reported. TNM classification is a cancer staging system. m, male; f, female.

Supplementary Table 11

	α	β	α	β	α	β
**Sézary Patient**	**Predominant *V***		**Predominant *J***		**Predominant CDR3**	
1	*TRAV26-1*	*TRBV12-5*	*TRAJ30*	*TRBJ1-5*	CIVRPYDRDDKIIF	CASSLGGTGHSNQPQHF
2	*TRAV4*	*TRBV4-3*	*TRAJ23*	*TRBJ1-6*	CLVGEPIYNQGGKLIF	CASSQDSPGPYNSPLHF
3	*TRAV12-2*	*TRBV29-1*	*TRAJ54*	*TRBJ2-7*	CAVSKGAQKLVF	CSVVGGPEAQYF
4	*TRAV8-2*	*TRBV18*	*TRAJ6*	*TRBJ1-5*	CVVSHASGGSYIPTF	CASSPSGDQPQHF
5	*TRAV13-1*	*TRBV4-3*	*TRAJ3*	*TRBJ1-2*	CAAFPYSSASKIIF	CASSQESWGANYGYTF
6	*TRAV14-DV4*	*TRBV19*	*TRAJ9*	*TRBJ2-1*	CAENPLNDYKLSF	CASSIAVSYNEQFF
7	*TRAV29-DV5*	*TRBV28*	*TRAJ43*	*TRBJ2-1*	CAGQLGNNNDMRF	CASSQGSGNQPQHF
8	*TRAV2*	*TRBV6-5*	*TRAJ42*	*TRBJ2-7*	CAVDIHLNYGGSQGNLIF	CASSSSGKGYEQYF
9	*TRAV2*	*TRBV7-3*	*TRAJ15*	*TRBJ2-3*	CAVALNQAGTALIF	CASSLAGTLTDTQYF
10	*TRAV20*	*TRBV19*	*TRAJ9*	*TRBJ1-4*	CAVQVETGGFKTIF	CASSISFRQTLNEKLFF

Supplementary Table 11. **Preferentially used *V* and *J* genes and predominant CDR3 amino acid sequence for each Sézary patient.** If a specific sequence or gene was found in more than one patient, it was highlighted in grey. Results indicate the occurrence of different predominating CDR3 sequences in every patient and, in few exception, the preferred usage of specific *V* or *J* genes in more than one individual. Results for both TCR chains are shown. Two replicates were performed for each sample and sequencing results were combined. *TRAV*, TCR α-chain variable gene; *TRAJ*, TCR α-chain joining gene; *TRBV*, TCR β-chain variable gene; *TRBJ*, TCR β-chain joining gene; CDR3, complementarity determining region 3; *V*, variable, *J*, joining.

Supplementary Table 12

a **α –Chain**

V Genes	Mouse	*V* Genes	Mouse
TRAV1		*TRAV9D-2*	
TRAV2		*TRAV9D-3*	
TRAV3-1		*TRAV9D-4*	
TRAV3-2		*TRAV9-1*	
TRAV3D-3		*TRAV9-2*	
TRAV3-3		*TRAV9-3*	
TRAV3-4		*TRAV9-4*	
TRAV4D-2-pseudogene		*TRAV10D*	
TRAV4D-3		*TRAV10*	
TRAV4D-4		*TRAV11D*	
TRAV4-1		*TRAV11*	
TRAV4-2		*TRAV12D-1*	
TRAV4-3		*TRAV12D-2*	
TRAV4-4-DV10		*TRAV12D-3*	
TRAV5-1		*TRAV12-1*	
TRAV5D-2-pseudogene		*TRAV12-2*	
TRAV5D-4-orf		*TRAV12-3*	
TRAV5-2-pseudogene		*TRAV12-4*	
TRAV5-4-orf		*TRAV13D-1*	
TRAV6-1		*TRAV13D-2*	
TRAV6-2		*TRAV13D-3*	
TRAV6D-3		*TRAV13D-4*	
TRAV6D-4		*TRAV13-1*	
TRAV6D-5		*TRAV13-2*	
TRAV6D-6		*TRAV13-3*	
TRAV6D-7		*TRAV13-4-DV7*	
TRAV6-3		*TRAV13-5*	
TRAV6-4		*TRAV14D-1*	
TRAV6-5		*TRAV14D-2*	
TRAV6-6		*TRAV14D-3-DV8*	
TRAV6-7-DV9		*TRAV14-1*	
TRAV7-1		*TRAV14-2*	
TRAV7D-2		*TRAV14-3*	
TRAV7D-3		*TRAV15-1-DV6D-1*	
TRAV7D-4		*TRAV15-2-DV6D-2*	
TRAV7D-5		*TRAV15D-3*	
TRAV7D-6		*TRAV15-1-DV6-1*	
TRAV7-2		*TRAV15-2-DV6-2*	
TRAV7-3		*TRAV15-3*	
TRAV7-4		*TRAV16D-DV11*	
TRAV7-5		*TRAV16*	
TRAV7-6		*TRAV17*	
TRAV8D-1		*TRAV18-orf*	
TRAV8D-2		*TRAV19*	
TRAV8-1		*TRAV20-pseudogene*	
TRAV8-2		*TRAV21-DV12*	
TRAV9D-1		*TRAV23*	

J Genes	Mouse	J Genes	Mouse
TRAJ2		*TRAJ32*	
TRAJ3-orf		*TRAJ33*	
TRAJ4-orf		*TRAJ34*	
TRAJ5		*TRAJ35*	
TRAJ6		*TRAJ36-pseudogene*	
TRAJ7-orf		*TRAJ37*	
TRAJ9		*TRAJ38*	
TRAJ11		*TRAJ39*	
TRAJ12		*TRAJ40*	
TRAJ13		*TRAJ41-orf*	
TRAJ15		*TRAJ42*	
TRAJ16		*TRAJ43*	
TRAJ17		*TRAJ44-orf*	
TRAJ18		*TRAJ45*	
TRAJ19-orf		*TRAJ46-orf*	
TRAJ20-orf		*TRAJ47-orf*	
TRAJ21		*TRAJ48*	
TRAJ22		*TRAJ49*	
TRAJ23		*TRAJ50*	
TRAJ24		*TRAJ52*	
TRAJ25-orf		*TRAJ53*	
TRAJ26		*TRAJ54-pseudogene*	
TRAJ27		*TRAJ56*	
TRAJ28		*TRAJ57*	
TRAJ29-orf		*TRAJ58*	
TRAJ30		*TRAJ59-orf*	
TRAJ31		*TRAJ60-pseudogene*	
		TRAJ61-pseudogene	

b **β –Chain**

V Genes	Mouse
TRBV1	
TRBV2	
TRBV3	
TRBV4	
TRBV5	
TRBV8-1-pseudogene	
TRBV9-pseudogene	
TRBV10-1-pseudogene	
TRBV12-1	
TRBV12-2	
TRBV13-1	
TRBV13-2	
TRBV13-3	
TRBV14-1	
TRBV15-1	
TRBV16-1	
TRBV17-1	
TRBV19-1	
TRBV20-1	
TRBV21-1-orf	
TRBV22-1-pseudogene	
TRBV23-1	
TRBV24-1	
TRBV24-2-pseudogene	
TRBV25-1-pseudogene	
TRBV26-1	
TRBV29-1	
TRBV30-1	
TRBV31-1	

J Genes	Mouse
TRBJ1-1	
TRBJ1-2	
TRBJ1-3	
TRBJ1-4	
TRBJ1-5	
TRBJ1-6-orf	
TRBJ1-7-orf	
TRBJ2-1	
TRBJ2-2	
TRBJ2-3	
TRBJ2-4	
TRBJ2-5	
TRBJ2-6	
TRBJ2-7	

Supplementary Table 12. *V* and *J* gene usage for alpha- and beta-chains in mouse. Functional genes, open reading frame (ORF) regions and pseudogenes are included. Grey shading denotes genes identified by TCR-LA-MC PCR sequencing. **a**, α-chain genes; **b**, β-chain genes. TCR-LA-MC PCR, TCR ligation anchored-magnetically captured PCR; *TRAV*, TCR α-chain variable gene; *TRAJ*, TCR α-chain joining gene; *TRBV*, TCR β-chain variable gene; *TRBJ*, TCR β-chain joining gene.

Supplementary References

1. Crooks, G.E., Hon, G., Chandonia, J.M. & Brenner, S.E. WebLogo: a sequence logo generator. *Genome research* **14**, 1188-1190 (2004).
2. Krzywinski, M. et al. Circos: an information aesthetic for comparative genomics. *Genome research* **19**, 1639-1645 (2009).
3. Freeman, J.D., Warren, R.L., Webb, J.R., Nelson, B.H. & Holt, R.A. Profiling the T-cell receptor beta-chain repertoire by massively parallel sequencing. *Genome research* **19**, 1817-1824 (2009).
4. Willenbrock K., Roers A., Seidl C., Wacker H.H., Küppers R., Hansmann M.L. Analysis of T-cell subpopulations in T-cell non-Hodgkin's lymphoma of angioimmunoblastic lymphadenopathy with dysproteinemia type by single target gene amplification of T cell receptor- beta gene rearrangements. Am J Pathol. **158(5)**:1851-7 (2001).
5. Bolotin, D.A. et al. Next generation sequencing for TCR repertoire profiling: platform-specific features and correction algorithms. *European journal of immunology* **42**, 3073-3083 (2012).
6. Bolotin, D.A. et al. MiTCR: software for T-cell receptor sequencing data analysis. *Nature methods* **10**, 813-814 (2013).
7. Porcelli, S., Yockey, C.E., Brenner, M.B. & Balk, S.P. Analysis of T cell antigen receptor (TCR) expression by human peripheral blood CD4-8- alpha/beta T cells demonstrates preferential use of several V beta genes and an invariant TCR alpha chain. *The Journal of experimental medicine* **178**, 1-16 (1993).
8. Greenaway H. Y., Ng B., Price D. A., Douek D. C., Davenport M. P. Venturi V., NKT and MAIT invariant TCRα sequences can be produced efficiently by VJ gene recombination. *Immunobiology* **218**, 213-224 (2013).
9. Robins, H.S. et al. Overlap and effective size of the human CD8+ T cell receptor repertoire. *Science translational medicine* **2**, 47ra64 (2010).
10. Price, D.A. et al. Avidity for antigen shapes clonal dominance in CD8+ T cell populations specific for persistent DNA viruses. *The Journal of experimental medicine* **202**, 1349-1361 (2005).
11. Lehner, P.J. et al. Human HLA-A0201-restricted cytotoxic T lymphocyte recognition of influenza A is dominated by T cells bearing the V beta 17 gene segment. *The Journal of experimental medicine* **181**, 79-91 (1995).
12. Trautmann, L. et al. Selection of T cell clones expressing high-affinity public TCRs within Human cytomegalovirus-specific CD8 T cell responses. *Journal of immunology (Baltimore, Md. : 1950)* **175**, 6123-6132 (2005).
13. Miles, J.J. et al. Genetic and structural basis for selection of a ubiquitous T cell receptor deployed in Epstein-Barr virus infection. *PLoS pathogens* **6**, e1001198 (2010).
14. Moss, P.A. et al. Extensive conservation of alpha and beta chains of the human T-cell antigen receptor recognizing HLA-A2 and influenza A matrix peptide. *Proc Natl Acad Sci U S A* **88**, 8987-8990 (1991).
